# Temperature and repeated catecholamine surges modulate regional wall motion abnormalities in a rodent takotsubo syndrome model

**DOI:** 10.1038/s41598-025-88410-9

**Published:** 2025-01-31

**Authors:** Ermir Zulfaj, AmirAli Nejat, Abdulhussain Haamid, Aaron Espinosa, Ahmed Elmahdy, Tetiana Pylova, Sandeep Jha, Björn Redfors, Elmir Omerovic

**Affiliations:** 1 Department of Molecular and Clinical Medicine, Institute of Medicine, Sahlgrenska Academy at Gothenburg University, Bruna Stråket 16, Gothenburg, 413 45 Sweden; 2 Department of Cardiology, Sahlgrenska University Hospital, Göteborg, Sweden; 3 Core Facilities - Experimental Biomedicine, Sahlgrenska Academy, Göteborg, Sweden

**Keywords:** Takotsubo Syndrome, Regional Wall Motion abnormalities, Rat model, Body temperature, Echocardiography, Complication, Catecholamine, Heart failure, Cardiomyopathies, Heart failure

## Abstract

Transient regional wall motion abnormalities (RWMA) after stress are a hallmark of experimental and clinical Takotsubo Syndrome (TS). The mechanisms driving RWMA remain incompletely understood. This study investigates these mechanisms in a small-animal model of TS, validating the model’s complication profile, recovery dynamics, and recurrence, while assessing the predictive value of echocardiography. Male, 7-9-week-old rats underwent TS induction via isoprenaline infusion. The effects of body temperature and repeated catecholaminergic surges on RWMA were assessed using high-resolution speckle-tracking echocardiography. Complications were evaluated through ECG, autopsy, and blood gas analysis. The TS phenotype showed reduced longitudinal strain with RWMA characterized by apical end-systolic akinesia/dyskinesia, a contracting base, and a narrow transition zone, resulting in apical ballooning. RWMA originated from the apex and recovered in the opposite direction. Hyperthermia increased RWMA incidence, while hypothermia attenuated RWMA (*p* = 0.008). A repeated catecholamine surge exacerbated RWMA in the acute phase (*p* = 0.042) but not during recovery (*p* = 0.308), with reduced RWMA susceptibility after recovery (RR 0.45, 95%CI; 0.27–0.76). Systolic function at initial echocardiography predicted worse outcomes (odds ratio 0.73; 95%CI 0.57–0.85), and RWMA extent correlated with recovery time (Rho = 0.772). TS rats developed complications similar to patients, including heart failure, arrhythmias, and thrombus formation. This study validates the small-animal TS model by replicating patient findings, and emphasizing the importance of controlling body temperature and limiting adrenergic drug use in managing TS. RWMA analysis offers promise as a diagnostic and prognostic tool in TS, and the delayed adaptive response presents a potential pathway for therapeutic intervention.

**Supplementary Information:**

The online version contains supplementary material available at 10.1038/s41598-025-88410-9.

## Introduction

Takotsubo syndrome (TS) resembles acute myocardial infarction in terms of mortality and clinical symptoms^1^. Clinicians recognize the syndrome by its reversible regional wall motion abnormalities (RWMA), often showing “apical ballooning” on imaging. While the exact mechanisms of TS remain unclear, it’s widely agreed that catecholamines play an important role. Human disease models are crucial for elucidating underlying mechanisms^2^. Our recently refined TS model accurately replicates key patient findings in both sexes, including apical ballooning, the natural course of acute onset and recovery within days, ECG changes, and clinically relevant preceding triggers^3^. While these features represent important advances in experimental TS research, a thorough investigation of both RWMA—the hallmark of this syndrome—and other clinical manifestations is essential for understanding condition development, progression, and resolution.

The release of catecholamines and the onset of TS are often triggered by stress. These triggers can be influenced by various factors, with studies suggesting a link between higher temperatures and increased TS incidence^4^. TS has relatively high complication rates, especially during the acute phase, with complications including heart failure, thrombus formation, and arrhythmia^5^. Several factors influence prognosis, including age, sex, comorbidities, and cardiac function^6,7^. While most patients recover contractile function within days to weeks^8^, the factors influencing recovery timing remain poorly understood. Recurrence occurs in 4–15% of cases^9,10^, with about 20% exhibiting a different TS pattern, suggesting a possible protective effect on previously affected region^11^.

In this article, we present findings that comprehensively validate our newly developed small-animal TS model by examining its complication profile, recovery pattern, recurrence, and the predictive value of echocardiography. Additionally, we use this model to explore various aspects of TS pathophysiology, including the impact of body temperature and a repeated catecholamine surge on RWMA development during different phases. We further define the RWMA in our model, identifying the necessary elements for the characteristic phenotype of apical ballooning and discuss its pathophysiological implications. We investigate the assessment of RWMA and systolic cardiac function after stress using various echocardiographic modalities. Additionally, we perform blood gas analysis to study physiological and metabolic changes during TS progression.

## Methods

### Animals and experimental Set-Up

**Fig. 1:**
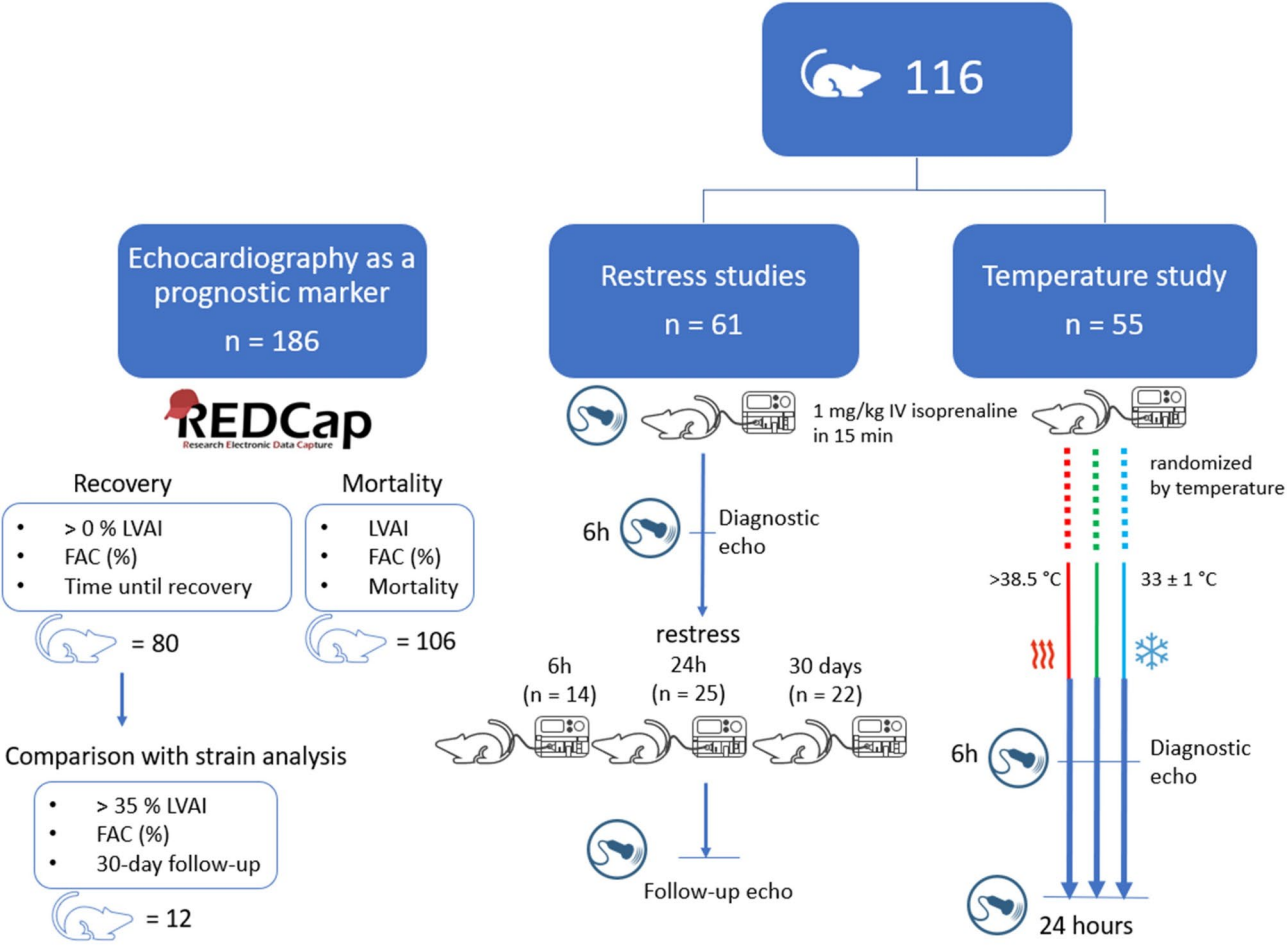
Summary of Experimental Designs. This figure provides an overview of the four experimental studies conducted. The investigation of RWMA and the predictive value of echocardiography utilized data from an established REDCap electronic database. Observations included data on akinesia extent (LVAI), systolic function (FAC), and recovery or mortality, with validation through strain analysis in a subset of cases. Restress studies evaluated the effects of a second catecholaminergic surge at intervals of 6 h, 24 h, or 30 days post-initial surge. The temperature study assessed the impact of body temperature on RWMA development. High-resolution speckle-tracking echocardiography was employed to assess heart function and wall motion abnormalities. Key study outcomes included mortality, incidence and extent of apical akinesia, and recovery, with complications examined via ECG, echocardiography, autopsy, and blood gas analysis.

All animal work adhered to national guidelines for experimental animal use, the study is reported in accordance with ARRIVE guidelines, and the study protocol received approval from the regional Animal Ethics Committee in Sweden (Dnr 5.8.18–02426/2023). At the end of the experiment, all animals were euthanized using isoflurane anaesthesia followed by heart excision, in accordance with approved ethical permit. Figure 1 provides a summary of our experimental designs. 116 outbred male nRjHan rats (Sprague Dawley, Janvier Labs, France), aged 7–9 weeks, were used. For summary statistics for each study group, refer to Tables 1 and 2. The rats were housed 2–3 per cage and acclimatized for a week in a temperature-controlled facility (19–21 °C) with a 12-hour light/dark cycle and free access to food and water. The animals were anesthetized intraperitoneally with ketamine (70 mg/kg) and midazolam (3.5 mg/kg). For TS induction, 1 mg/kg of isoprenaline was infused over 15 min via the tail vein using an infusion pump^3^.

**Table 1:** Summary animal characteristics.

	30d restress			24 h restress		6 h restress		Temperature Study
	baseline (*N* = 22)	30 days (*N* = 22)		baseline (*N* = 25)		baseline (*N* = 14)		baseline (*N* = 55)
Weight (kg)								
Mean (SD)	0.343 (0.10)	0.468 (0.08)		0.400 (0.03)		0.420 (0.02)		0.331 (0.02)
Median [Min, Max]	0.318 [0.29, 0.66]	0.445 [0.39, 0.70]		0.398 [0.35, 0.47]		0.417 [0.38, 0.46]		0.327 [0.29, 0.39]
Age (days)								
Mean (SD)	55.1 (13.9)	81.8 (16.1)		59.1 (2.20)		59.7 (1.82)		47.7 (1.31)
Median [Min, Max]	49.0 [47.0, 97.0]	79.0 [68.0, 127]		60.0 [56.0, 61.0]		59.5 [58.0, 62.0]		48.0 [46.0, 50.0]
Body Temperature (°C)								
Mean (SD)	37.5 (0.349)	37.5 (0.402)		37.6 (0.526)		37.4 (0.727)		37.0 (0.975)
Median [Min, Max]	37.5 [37.0, 38.1]	37.5 [36.8, 38.5]		37.6 [36.6, 38.6]		37.5 [36.2, 38.3]		37.2 [33.0, 38.3]
Heart Rate (bpm)								
Mean (SD)	361 (57.5)	318 (39.0)		380 (65.6)		344 (50.4)		416 (46.8)
Median [Min, Max]	360 [276, 470]	310 [255, 390]		370 [280, 515]		340 [270, 445]		410 [322, 505]
SpO2 (%)								
Mean (SD)	93.7 (6.51)	94.1 (5.61)		-		94.9 (5.90)		95.5 (5.86)
Median [Min, Max]	97.5 [84.0, 99.0]	95.0 [83.0, 99.0]		-		99.0 [86.0, 99.0]		99.0 [75.0, 99.0]
Sex								
Male	22 (100%)	22 (100%)		25 (100%)		14 (100%)		55 (100%)

Summary statistics of each study at baseline prior induction of takotsubo syndrome (TS). The restress studies evaluated the effect of a second stressor, an isoprenaline challenge, at various time points corresponding to different phases of TS: 30 days (post recovery), 24 h (during recovery), 6 h (peak of apical ballooning). The temperature study evaluated the influence of body temperature on the development of TS.

**Table 2:** Summary animal characteristics.

	Mortality prediction (*N* = 106)	Recovery prediction (*N* = 80)
Weight (kg)		
Mean (SD)	0.328 (0.0223)	0.321 (0.0214)
Median [Min, Max]	0.330 [0.279, 0.374]	0.321 [0.275, 0.372]
Age (days)		
Mean (SD)	47.6 (1.62)	47.4 (1.91)
Median [Min, Max]	48.0 [43.0, 51.0]	47.0 [43.0, 55.0]
Body Temperature (°C)		
Mean (SD)	37.3 (0.397)	37.2 (0.310)
Median [Min, Max]	37.2 [36.5, 38.3]	37.2 [36.6, 38.0]
Heart Rate (bpm)		
Mean (SD)	448 (62.9)	426 (48.5)
Median [Min, Max]	438 [341, 582]	426 [337, 545]
SpO2 (%)		
Mean (SD)	97.5 (3.16)	96.4 (4.98)
Median [Min, Max]	99.0 [87.0, 99.0]	99.0 [77.0, 99.0]
Sex		
Male	106 (100%)	80 (100%)
Iso dose (mg/kg)		
Mean (SD)	3.82 (2.76)	6.25 (0)
Median [Min, Max]	6.25 [0.500, 6.25]	6.25 [6.25, 6.25]
Infusion time (min)		
Mean (SD)	18.3 (15.9)	19.5 (20.2)
Median [Min, Max]	15.0 [1.00, 60.0]	15.0 [1.00, 60.0]
FAC (%)		
Mean (SD)	40.3 (16.1)	33.2 (9.11)
Median [Min, Max]	42.9 [6.92, 71.7]	33.3 [18.8, 51.6]
LVAI (%)		
Mean (SD)	15.7 (13.3)	25.6 (10.1)
Median [Min, Max]	15.3 [0, 45.5]	25.0 [5.98, 48.0]
Recovery (hours)		
Mean (SD)	60.5 (37.1)	60.9 (38.4)
Median [Min, Max]	48.0 [0, 216]	48.0 [24.0, 216]
Missing	60 (56.6%)	0 (0%)
Deaths		
0	91 (85.8%)	80 (100%)
1	15 (14.2%)	0 (0%)

Summary statistics at baseline prior TS induction. LVAI and FAC are derived from first echocardiography post induction. Iso dose; total dose of isoprenaline. Infusion time; duration of isoprenaline infusion.

### High-resolution echocardiography

Cardiac function and akinesia were evaluated in lightly anesthetized rats using previously described echocardiographic methods^3^. We estimated global left ventricular (LV) systolic function in the parasternal long axis (LAX) view using fractional area change (FAC)^12^ and the extent of akinesia using left ventricular akinesia index (LVAI) (Supplementary Video 1)^13^. Total fractional shortening (FS, %) was assessed in the parasternal short axis (SAX) view (Supplementary Video 2). End-systolic and diastolic volumes, ejection fraction (EF, %), and stroke volume was derived from the SAX images (Supplementary Fig. 1). The wall motion score index (WMSI) was visually assessed on a scale of 1–3 (normal or hyperkinetic, hypokinetic, akinetic or dyskinetic) using the 17-segment model in SAX^14,15^. The 17th segment was binary scored (akinetic or normal) based on apical akinesia in LAX. Data acquisition and subsequent analysis were performed using FUJIFILM VisualSonics Inc.‘s Vevo Lab 5.6.0 software, with group assignments blinded to the researchers. TS incidence was defined as any visible akinetic segment in LAX extending circumferentially (Supplementary Fig. 1), with recovery indicated by the absence of akinetic segments. We reconstructed 3D images by delineating the endocardium in a series of SAX view images, with a 150 μm increment from the aortic root to the apex during diastole and systole. The resulting diastolic and systolic volumes were overlaid for visualization.

### Speckle-tracking echocardiography

Longitudinal and segmental radial strain were measured from at least two consecutive cardiac cycles in the LAX view of LV at ≥ 120 frames/s using Vevo Lab 5.6.0 software. To ensure data quality, cardiac cycles affected by respiratory artefacts were excluded. We delineated the endocardium with 48 sampling points over a 19–28 mm length, spaced at 400–580 μm intervals, and divided the LV into six segments: Base, mid, and apex (posterior and anterior). Strain—the length change during myocardial contraction and relaxation— was analysed and averaged for peak radial strain (%) in each segment (base, mid, apex). End-systolic radial strain, peak-systolic radial strain, post-systolic radial strain (peak radial strain occurring in the post-systolic phase), and time-to-peak radial strain were also assessed, providing a comprehensive understanding of each segment’s contractility and synchronicity (Supplementary Fig. 2). We established a threshold for akinesia by assessing the FS and averaged peak radial strain of both apical and basal segments in a scatter plot. This threshold was used in the strain analysis to measure the akinetic region (strain-derived akinetic region) at the moment of aortic valve closure (Supplementary Fig. 2). As indicated by the dV/dt-graph, the initiation of isovolumetric relaxation helped identify aortic valve closure. Longitudinal strain (LS, %) was computed using the strain formula applied to the entire endomyocardial line (Supplementary Video 3).

### LVAI and FAC as markers in TS

One of our aims was to investigate the predictive value of echocardiography in our TS model, specifically predicting recovery and mortality using systolic cardiac function or the extent of wall motion abnormality (LVAI). While developing our refined TS model^3^, we established a database using REDCap electronic data capture tools. From this database, we collected observations that included echocardiographic data on LVAI and FAC, along with mortality or time until recovery, for the prediction models (Table 2). All rats underwent intravenous induction with isoprenaline, although total dose (0.5–6.25 mg/kg) and infusion time (1–60 min) varied.

In a subgroup of these images, strain analysis was performed to further evaluate and define the RWMA and apical ballooning phenotype. We also compared FAC, EF, and LS as measures of cardiac function, alongside comparing visual assessment with strain-derived measurements for the akinetic region. We included all rats with at least 35% LVAI in the acute phase that underwent continuous 30-day imaging. We included additional rats without the TS phenotype for the FAC and LS comparison. By assessing the WMSI on these subgroups of images, we visualized the recovery pattern in our TS model.

### The effect of a second catecholaminergic surge

We aimed to evaluate the effect of a second catecholaminergic surge during different phases of TS and whether a history of TS or a previous episode of catecholaminergic surge without TS would change the propensity after 30 days. A second dose of 1 mg/kg isoprenaline was administered intravenously over 15 min to rats either 6 h (*n* = 14), 24 h (*n* = 25), or 30 days (*n* = 22) after the first dose.

### Blood gas analysis and complication profile

Complications were examined via ECG, echocardiography, blood gas analysis, and autopsy. Blood gas analysis was performed on 11 rats using the epoc^®^ Blood Analysis System^16^ (Siemens Healthineers) at baseline just before TS induction and then at 30 min, 90 min, 6 h, and 24 h after induction. We analysed single lead I ECG recordings from 131 rats involved in the infusion time study from our previous work^3^. These recordings, which monitored TS induction for at least 60 min or until death, were manually reviewed to detect significant arrhythmias, including ventricular tachycardia (VT), ventricular fibrillation (VF), and third-degree atrioventricular block (AV-block). VT was defined as more than four consecutive ventricular complexes, including polymorphic VT and Torsade de Pointes. VF was defined as a non-progressive variation of the shape, peak-to-peak, and height of the ventricular complexes. We classified each part in complex arrhythmias, where different types segued into one another without interruption. For example, we observed that all episodes of VF were preceded by VT, but not all episodes of VT degenerated into VF.

### The role of body temperature in TS development

Since our previous TS model, which utilized high-dose intraperitoneal isoprenaline, required high body temperatures to develop the TS phenotype^12,17^, we wanted to evaluate the effect of body temperature in our refined model. Fifty-five rats underwent TS induction and were randomized to one of three temperature groups during the first 60 min: hyperthermia, normothermia, or hypothermia. The rats were kept on a heating pad set to 38 °C for the hyperthermic group with no cooling applied. For the normothermic and hypothermic groups, the rats were kept on a turned-off heating pad (21 °C), with ice packs applied as needed to maintain body temperature of 37.5 ± 0.5 °C and 33 ± 1 °C, respectively. For a subgroup (*n* = 2 per group), systemic blood pressure was measured in the common carotid artery using a 1.9 F P/V catheter in a pressure-only setting (ADV500, Transonic Scisense) connected to a PowerLab 8/35 data acquisition system (LabChart pro, Transonic Scisense).

### Statistical analysis

Randomization was blinded to the researchers and uploaded to the REDCap electronic data capture tools^18^, where all study data was collected. Data analyses were conducted using R 4.2.0, IBM SPSS 27, and InVivo Stat 4.2.0. Sample size determination, based on a power of 0.8, effect size η² = 0.19, and alpha of 0.05, targeted akinesia as the primary endpoint. A comprehensive overview of the statistical details is provided in the Supplementary File.

## Results

### Wall motion abnormalities in TS

**Fig. 2:**
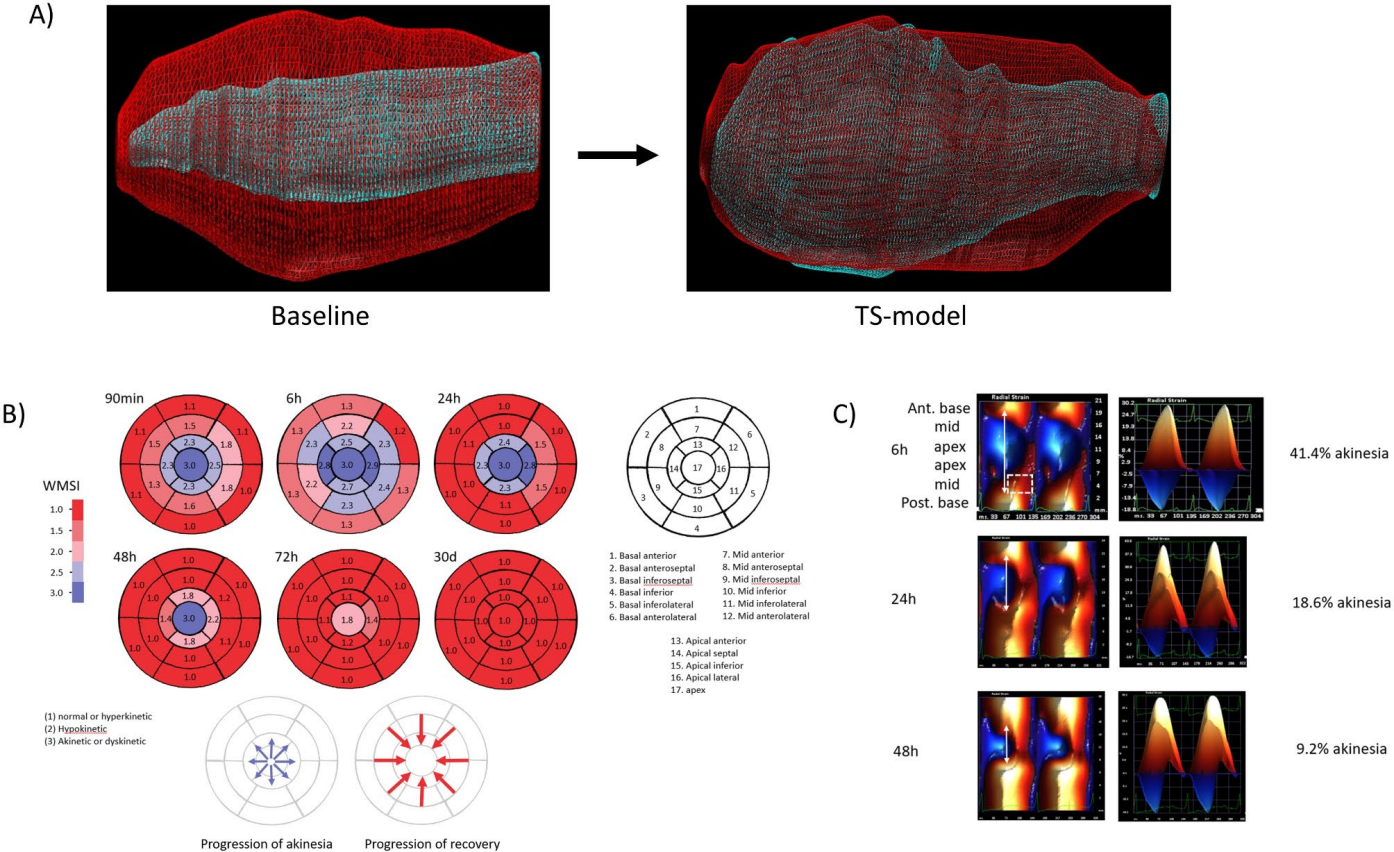
Development and Recovery of Apical Ballooning in Rats. (**A**) The animal model represented with diastolic and systolic 3D-volume overlay. Baseline on the left and the TS phenotype on the right, 6 h after stress. (**B**) Bulls eye map with 17 myocardial segments and their WMSI (mean) during the development and resolution of TS. While the RWMA is circumferential and originating from the apex, the recovery progresses in the opposite direction. *n* = 12. (1) normal or hyperkinetic, (2) hypokinetic, (3) akinetic or dyskinetic. (**C**) Representation of the TS recovery using heat maps of radial strain values taken in long-axes view, going from anterior base to posterior base. Double headed arrows highlight the extent of RWMA with its corresponding LVAI. Post-systolic strain is highlighted in the posterior mid segment (dashed box), which normalises at 24 h.

**Fig. 3:**
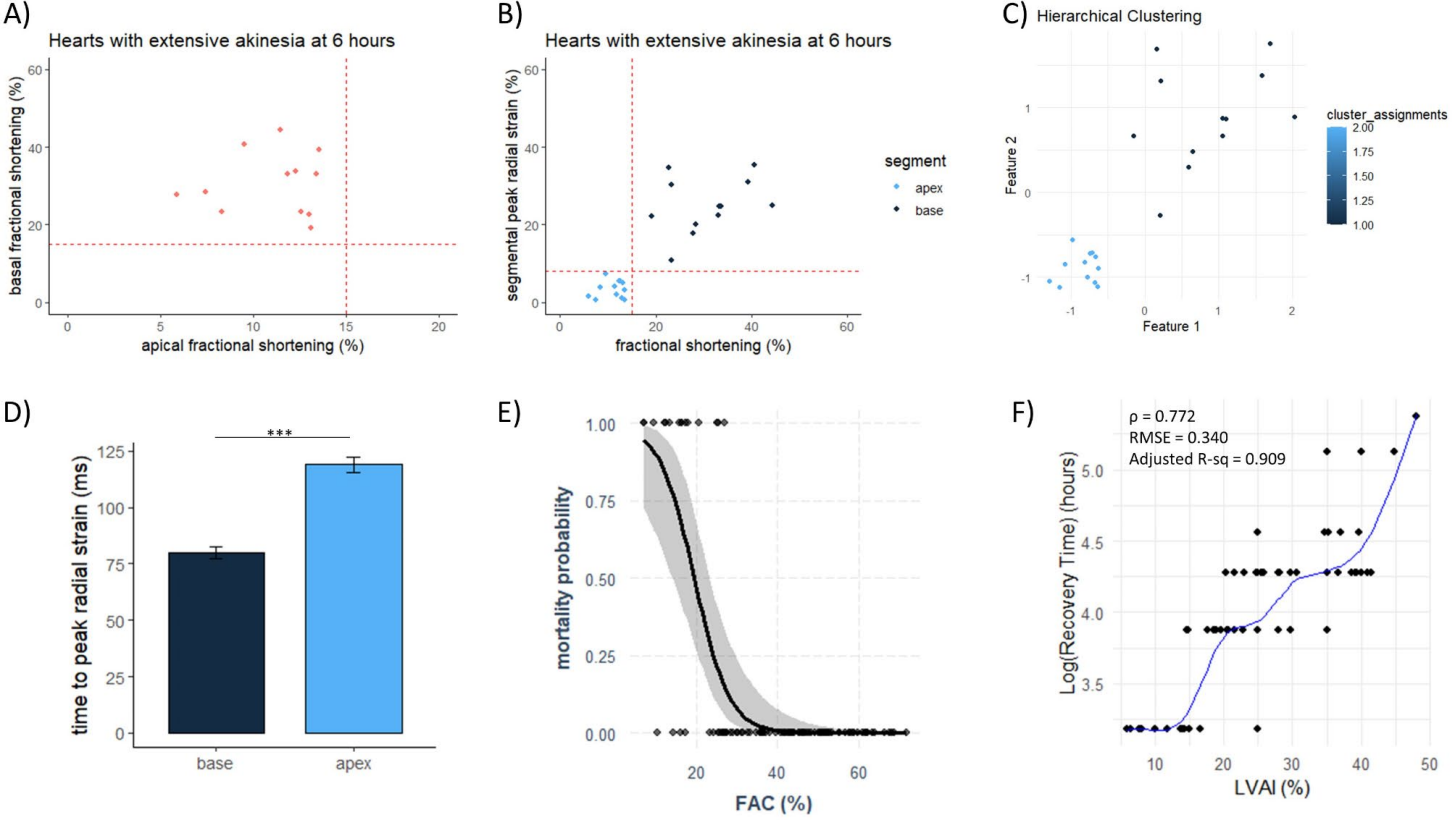
RWMA in TS and Echocardiography Demonstrate Predictive Value Within the Model. (**A**) Basal contraction varied in rats with extensive apical ballooning, but none dropped below 15% FS. Similarly, global hypokinesia cases (Supplementary Fig. 6C) stayed above this threshold. (**B**) A plot comparing FS and segmental peak radial strain at the base and apex in rats with pronounced TS shows a clear data separation, corresponding to a peak radial strain of 5–10% and FS < 15% (lower left quadrant). (**C**) Hierarchical clustering confirmed the underlying data structure. (**D**) Time to peak radial strain in apical and basal segments at 6 h post-TS. Paired t-test, *n* = 12. ***; *P* < 0.001. (**E**) Lower systolic function, measured by FAC from the first echocardiography post-TS induction, correlated with higher mortality. Odds ratio 0.79; 95% CI 0.69–0.87; *n* = 106. (**F**) LVAI strongly correlated with recovery time (*r* = 0.772). The WLS GAM model predicted recovery time based on LVAI with a cross-validated RMSE of 0.340 and an adjusted R-squared of 0.909; *n* = 80. *p* < 0.001.

Takotsubo syndrome and the development of akinesia originated from the apex and peaked after 4–7 h. At 6 h, the characteristic circumferential pattern, considered a hallmark of TS, was present (Fig. 2A, Supplementary Video 2 & 3). The TS phenotype exhibited reduced longitudinal strain and apical akinesia, with or without opposite LV deformation (i.e., dyskinesia) (Supplementary Fig. 2). While the development originated from the apex, recovery progressed in the opposite direction (Fig. 2B-C, Supplementary Fig. 3). Heat maps depicting the resolution of TS using speckle-tracking (Fig. 2C), showed that post-systolic strain was present prior the RWMA resolution. While the basal segments were synchronized with relatively preserved contraction, the akinetic mid segments, which recovered faster, exhibited a higher magnitude of post-systolic strain compared to the dyskinetic apical segments, which also exhibited post-systolic strain (Fig. 3D, Supplementary Figs. 3–4).

When comparing the peak radial strain and fractional shortening of basal and apical segments, we noted a separation of data points (Fig. 3A-C). A peak radial strain of 5–10% and/or a fractional shortening of < 15% was deemed as akinetic. The variation between peak radial strain and fractional shortening was initially low, with favourable agreement (Supplementary Fig. 5C-D, Rho = 0.846). The visual assessment of akinetic region correlated well with the akinetic region measured using strain analysis, using a cut-off value of 5% at the time of aortic valve closure (Supplementary Fig. 5E-F, Rho = 0.778). A cut-off value of 10% showed similar results (Rho 0.768). The transition area from akinetic to non-akinetic tissue ranged between 460 and 1750 μm (Supplementary Fig. 2).

### LVAI and FAC have predictive value in this preclinical TS model

A total of 106 observations with available echocardiographic data on LVAI and FAC, as well as mortality were identified. Eighty observations included data on the time-point for recovery and LVAI > 0%. The observations consisted of male rats, approximately 7 weeks of age (Table 2). Logistic regression analysis found that systolic cardiac function at the first echocardiography (2–6 h) was a good predictor for mortality (odds ratio 0.79; 95% confidence interval 0.69–0.87, Fig. 3E). The corresponding ROC curve can be found in supplementary material (Supplementary Fig. 6). The LVAI strongly correlated with time until recovery (ρ = 0.772, Fig. 3F). The fitted model for predicting recovery based on LVAI was robust, with an RMSE of 0.340, demonstrating a strong fit to the data (adjusted R-squared = 0.909). Systolic function, measured as FAC, had a robust correlation with LS (*r* = 0.796), although with a proportional bias (Supplementary Fig. 5A-B). We found that FAC was inversely associated with LVAI (rho = -0.898) and had a very strong correlation with EF (*r* = 0.914, Supplementary Fig. 6B). The cardiac function among the rats with no RWMA development (0% LVAI) post-stress varied, ranging from an increase to staying unchanged or even a decrease (Supplementary Fig. 6C).

### Repeated stress is detrimental in TS, while initial stress triggers adaptive protective responses

**Fig. 4:**
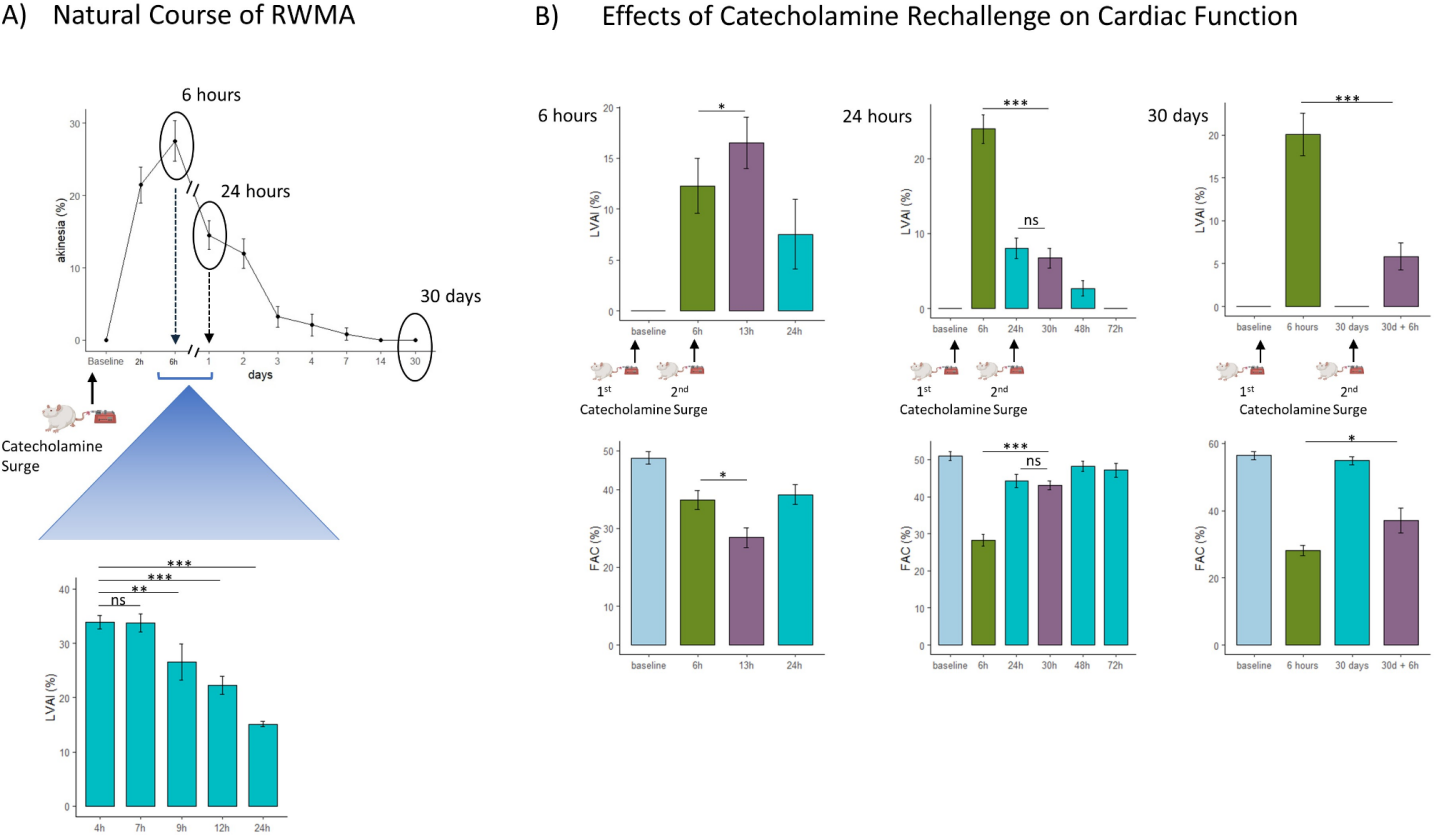
Second Catecholaminergic Surge Effects on RWMA During Different TS Phases. (**A**) The natural course of LVAI over 30 days with 6 h, 24 h, and 30d highlighted. Recovery begins at 9 h post-induction, and all rats recover in their contractile function before day 30. Mean ± SE. (**B**) A second isoprenaline challenge administered 6 h after the first, during peak LVAI, significantly increased LVAI. Conversely, a second dose during the recovery phase, 24 h later, did not significantly increase LVAI. Similarly, rats rechallenged after 30 days, representing those recovered from TS, showed smaller RWMA. Data are shown as mean ± SE, *n* = 14, 24, 22. Paired t-tests and one-way repeated measures ANOVA with Greenhouse-Geisser correction were used. P-values were adjusted using Bonferroni correction. ns; not significant. *; *P* < 0.05. **; *P* < 0.01. ***; *P* < 0.001.

In our previous work^3^, we illustrate the natural course, showing that recovery commences at 24 h, with the majority of rats recovering from RWMA within 3 days. Figure 4A further details the recovery timeline, showing its initiation as early as 9 h. An additional isoprenaline surge 6 h after the initial challenge worsened cardiac function and increased extent of RWMA (*p* = 0.042, Fig. 4B). Three rats did not develop any akinesia following the first challenge, but two of them developed after the second (Supplementary Fig. 7A). The additional isoprenaline infusion led to further LV dilation at 13 h, with increased end-diastolic volume and reduced cardiac function (Supplementary Fig. 8). End-systolic volume increased, reflecting worsened contractility. Heart rate showed no differences between echocardiographic time points, and both stroke volume and cardiac output remained unchanged (Supplementary Fig. 8). Conversely, an additional isoprenaline challenge after 24 h had no impact on the cardiac function or RWMA. All rats displayed a reduced LVAI after the second stress compared to the first (Supplementary Fig. 7B). Similarly, rats receiving the second stress 30 days later showed a reduced likelihood of developing the TS phenotype (*p* < 0.05, RR 0.45, 95%CI; 0.27–0.76, Supplementary Fig. 7C-D). Additionally, two rats did not exhibit any akinesia following the initial challenge, but one of them developed akinesia during the second round. No deaths occurred in the studies.

### Blood gas analysis of TS model

The TS model exhibited normal blood gas at the 6-hour time point, corresponding to the peak of akinesia in TS rats. However, during the induction phase, metabolic acidosis accompanied by an elevation in lactate levels was noted, which was subsequently compensated for both metabolically and respiratorily (Supplementary Table 1). These changes in blood parameters were observed in all rats that received isoprenaline infusion, irrespective of the development of RWMA. Interestingly, during this period, the rats that did not develop any akinesia exhibited higher glucose levels compared to the TS rats (Supplementary Fig. 6D). Other than blood glucose, no significant differences were observed in the blood gas analysis at different time points between rats that developed extensive akinesia and those that did not. Two rats that died failed to compensate for the metabolic acidosis, exhibiting respiratory insufficiency, kidney failure, and electrolyte imbalances.

### Complications reproduced as seen in human patients

**Fig. 5:**
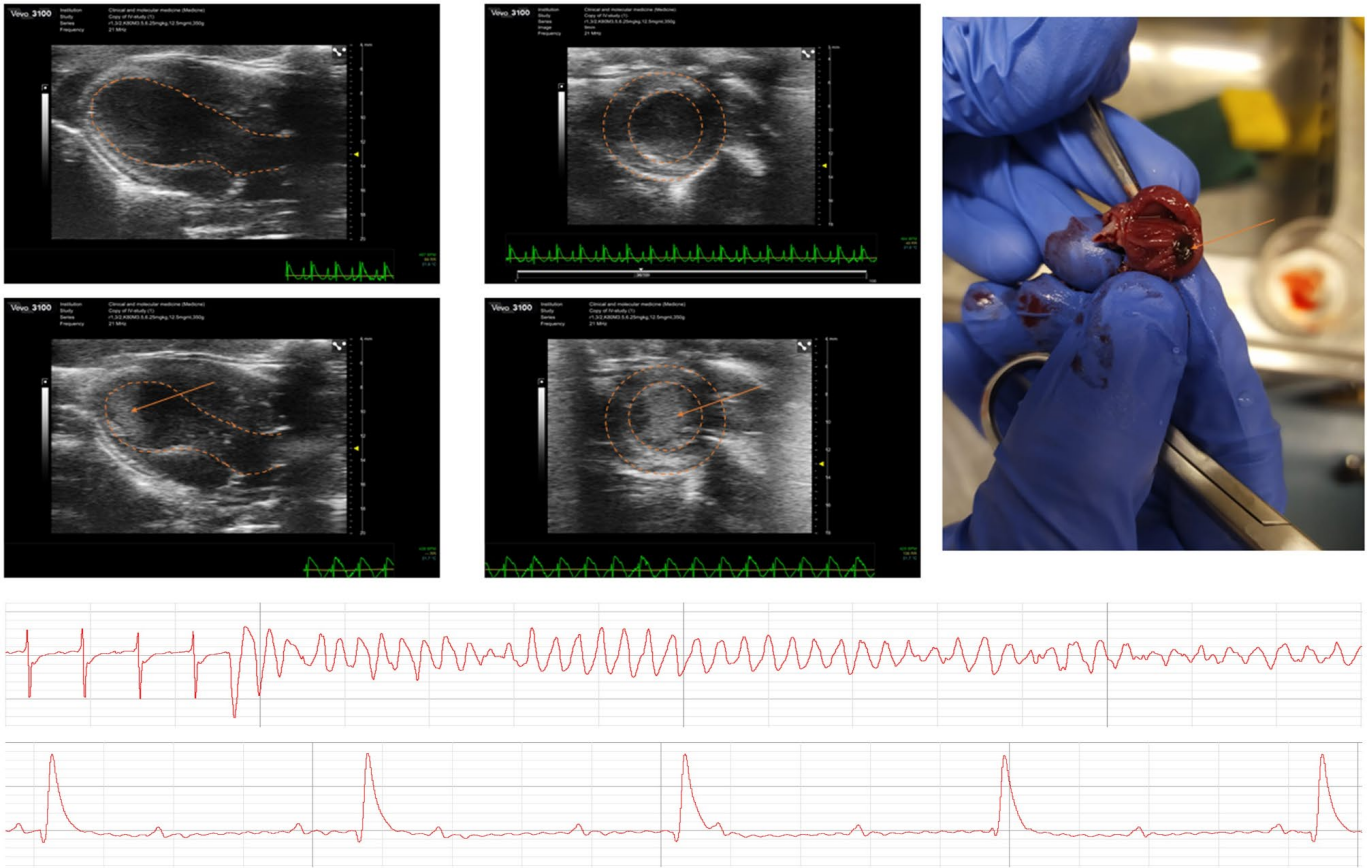
Complications Reproduced in TS Model. (**A**) Thrombus formation is shown in echocardiographic images taken 8 h post-induction (bottom) in the LAX view, showing visible apical ballooning (bottom left). This was confirmed in the SAX view, 9 mm below the mitral valve (bottom right), and via post-mortem analysis (right). Echocardiographic images from 3 h post-induction (top) show extensive apical ballooning without thrombus. The endo-/epicardium (dashed line) and thrombus (arrow) are highlighted for clarity. (**B**) Ventricular arrhythmia occurred early in the induction phase, and was the second most frequent cause of death after acute heart failure. (**C**) Atrioventricular blockage of varying degrees was observed. Here, a third-degree blockage is shown, marked by the total disconnection between atria and ventricles.

The diseased animals in our model exhibited several complications similar to those observed in TS patients, including heart failure, malignant arrhythmias, thrombus formation, bradyarrhythmia, and cardiogenic shock (Fig. 5, Supplementary Fig. 9). Among these complications, heart failure, defined as FAC lower than 45%, was the most frequent (84%). Some rats appeared to develop LV outflow tract obstruction, but we were unable to assess this due to the applied imaging protocol. Apical thrombus formation was observed only in rats with the development of apical ballooning phenotype (Fig. 5, supplementary video 4). Out of 213 cases in our REDCap data base with LVAI > 0% and an echocardiography performed at least 5 h after induction, 1 developed mural thrombus, and 2 developed a protruding thrombus in the apex (incidence 1.4%). From the ECG analysis, 11 out of 131 rats (8.4%) developed significant arrhythmia (Supplementary Fig. 9), with an hourly incidence rate of 0.155. The most common arrhythmia was VT (Supplementary Table 2). Both AV-block and VF were highly associated with death. The first 20 min of TS induction were identified as a particularly sensitive phase for arrhythmia occurrence, though arrhythmias were observed at later time points as well (Supplementary Fig. 9B). Out of the 131 rats, arrhythmias, including AV-block and VF, were identified as the cause of death in 7/38. The remaining 31/38 deaths displayed signs of severe heart failure as the most probable cause: hypoperfusion observed through a gradual decline in saturation and pallor of mucous membranes, lethargy, marked reduction in systolic cardiac function, and acidosis. The mortality rate across the four experimental studies was 4.3% (5 out of 116).

### Body temperature is an important determinant for the development of TS

**Fig. 6:**
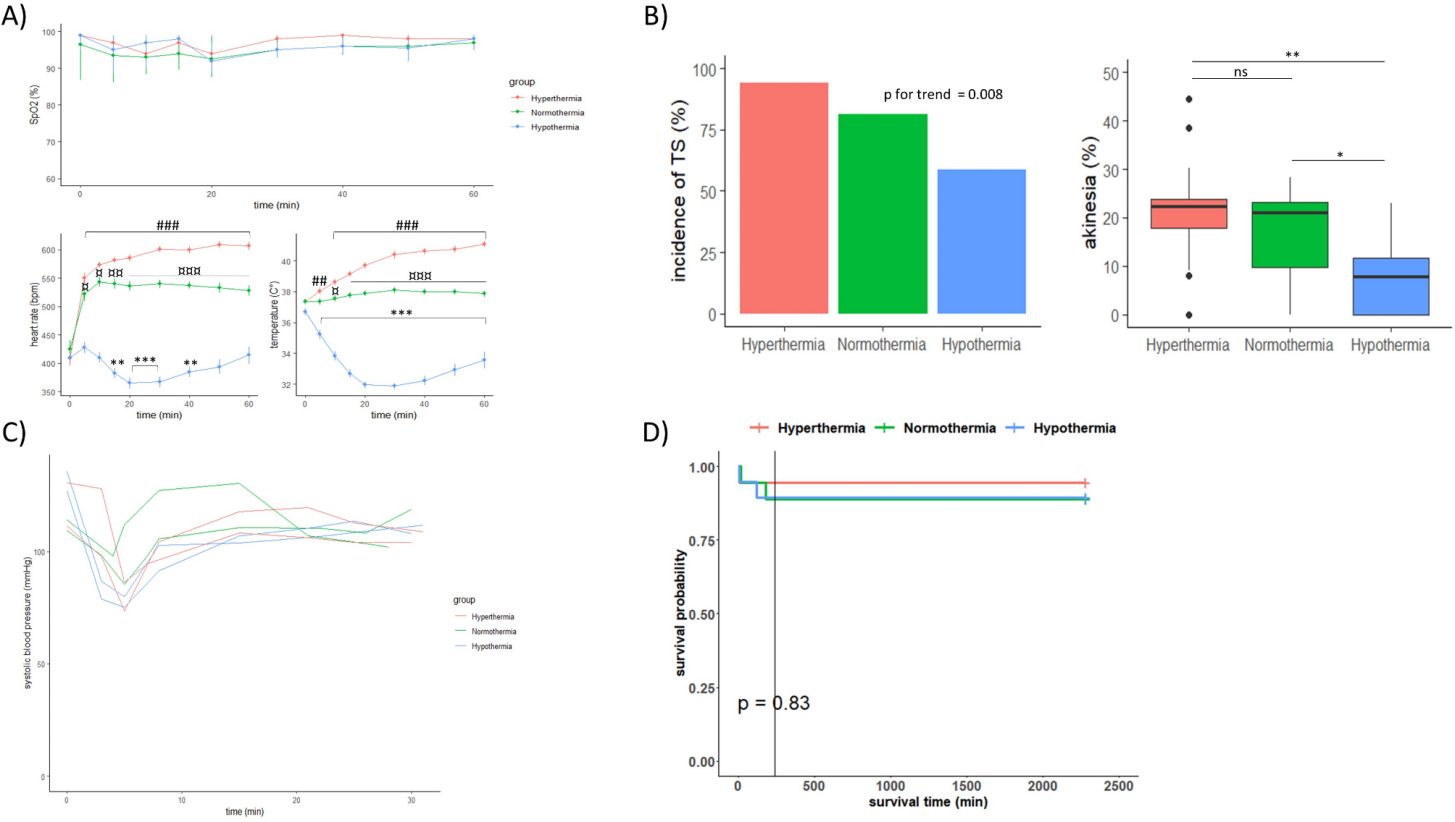
Body Temperature as a Determinant of TS Development. (**A**) SpO2 (median [Q1-Q3]), heart rate (mean + SE), and temperature (mean + SE) during the first hour of infusion. Data were analysed using two-way repeated measures ANOVA, with Bonferroni-adjusted P-values, *n* = 18 per group. *P < 0.05, **P < 0.01, ***P < 0.001 vs. baseline (hypothermia); ¤*P* < 0.05, ¤¤*P* < 0.01, ¤¤¤*P* < 0.001 hyperthermia vs. normothermia; #*P* < 0.05, ##*P* < 0.01, ###*P* < 0.001 vs. baseline (hyperthermia). (**B**) TS incidence increased with temperature in a stepwise fashion. Extended Mantel-Haenszel chi-square p for trend = 0.008. The hypothermic group had significantly lower LVAI at 4 h compared to the hyperthermic and normothermic groups, which did not differ significantly. Kruskal-Wallis test; ns ≥ 0.05, *P < 0.05, **P < 0.01. (**C**) Systolic blood pressure showed a consistent pattern across all groups, with an initial drop that later normalized. *n* = 2/group. (**D**) Mortality was 9.3%. Log-rank test; *P* = 0.83.

An increase in heart rate occurred soon after the start of isoprenaline and was highly dependent on body temperature (Fig. 6A). LVAI and TS incidence decreased in the hypothermic group, while it increased with higher body temperatures (Fig. 6B, *p* = 0.008). All temperature groups displayed a similar pattern in blood pressure measurements (Fig. 6C), showing an initial drop that later normalized. Mortality was low in all groups (Fig. 6D). The sudden drop in blood pressure occurred in conjunction with an increased heart rate, a drop in SpO2, and ECG changes.

## Discussion

Our objective was to conduct an in-depth study of RWMA, both defining it and investigate mechanisms driving its development, and further verify the usefulness of the small-animal model with a TS-like phenotype in rats for preclinical research. This represents the first comprehensive investigation to show a TS model accurately replicating human TS, including the RWMA, complication profile, recovery pattern, recurrence, and predictive value of cardiac function. We report three further significant discoveries: (1) body temperature plays a crucial role in the onset of RWMA following stress, (2) ongoing stress or catecholamine administration negatively impacts RWMA, and (3) adaptive mechanisms can lessen the occurrence of RWMA after post-stress conditions.

Stress induces various systemic and cardiac-specific adaptations^2^. After a catecholamine surge, we observed changes in myocardial contractility and timing, with a distinct separation between apical and basal segments. The characteristic apical ballooning in our model, as observed in TS patients, resulted from a combination of (1) akinetic or dyskinetic apical segments, (2) a contracting base (hyper-, normo-, or hypokinesia), and (3) a narrow transition zone. A dyssynchronous heart with basal contraction and apical akinesia or dyskinesia at aortic valve closure produces a similar phenotype independent of post-systolic strain. Conversely, restoring the synchrony would theoretically alleviate the phenotype in cases with preserved, but delayed, contractility. These novel findings indicate that RWMA in our TS model is shaped by contractile function and timing. Post-systolic strain is prevalent in the apical segments of TS patients, but its significance remains unclear^19^. Previous studies in animal models of myocardial infarction have linked the magnitude of post-systolic strain to recovery^20,21^. In this context, our findings suggest that post-systolic radial strain could indicate viable myocardium and help explain recovery rates in TS. The narrow transition zone underscores the heart’s region-specific susceptibilities, revealing distinct differences in myocardial response to catecholamine surges. A sharp disparity in contractility between adjacent regions, especially with a hypokinetic basal segment, could worsen cardiac output. At the same time, a hyperkinetic base may lead to LV outflow tract obstruction and increased wall stress, potentially causing injury^22^. This stress-induced transition zone challenges the receptor-mediated RWMA hypothesis. No evidence in humans or animals suggests a receptor density gradient sharp enough to explain these transition zones. A toxic- or receptor-mediated mechanism would likely produce diffuse rather than localized myocardial dysfunction. The presence or absence of this transition zone may indicate different TS subtypes or underlying mechanisms^2^.

Our analysis of strain and WMSI shows that akinesia begins at the apex, with recovery moving in the opposite direction—a pattern never previously described in a TS model but also observed in patients with apical ballooning^23^. These findings imply that apical regions are more vulnerable, with a gradient in severity and recovery mechanisms across the LV. We also found systolic cardiac function to be a predictor of poor outcomes, a feature previously known in human TS^6^ but now demonstrated for the first time in an experimental model. Additionally, we discovered a strong correlation between RWMA extent and recovery time—a novel finding never before reported in either clinical or experimental settings.

We previously demonstrated that the TS phenotype and LVAI are dose- and time-dependent on catecholamines^3^. Here, we show that recurring catecholaminergic surges in an established TS phenotype can worsen RWMA and cardiac function, emphasizing the need to limit adrenergic drug use in TS^5^. This is supported by observational studies showing that inotropic drugs increase short-term mortality tenfold^24,25^. Moreover, if RWMA extent predicts recovery, inotropic drugs could worsen cardiac function and delay recovery. The second isoprenaline surge further increased end-diastolic volume in our model, suggesting a compensatory response to balance the systolic dysfunction. While similar volume changes have been documented in stable TS patients^26^, a more comprehensive and temporal analysis of hemodynamics in the TS model is needed. Patients with a history of TS have a recurrence rate of 4–15%^9,10^. Our model effectively reproduces this phenomenon, demonstrating recurrence of the TS phenotype in rats rechallenged after 30 days, albeit in a controlled experimental setting. No other TS model has shown this feature. Interestingly, a repeated catecholaminergic surge during recovery at 24 h did not worsen cardiac function or RWMA, suggesting a delayed adaptive response that limits future RWMA post-stress. This reduced susceptibility, though less evident, persisted 30 days post-recovery. However, the mechanism underlying this adaptive response remains unclear. Histological analysis shows no significant changes until 24 h after isoprenaline administration, when cell infiltration and necrosis become evident^3^, suggesting a possible role of immunological mechanisms. Other mechanisms, such as extracellular vesicles acting as mediators of cardioprotection^27^, also deserve investigation. It is uncertain whether a longer waiting period (e.g., > 30 days) would restore TS susceptibility in our model. Further research is required to understand this adaptive response and to determine whether similar adaptations occur in humans.

The transient metabolic acidosis observed in blood gas analysis is likely due to the direct effect of catecholamine, which overstimulates glycolysis and leads to lactate production. While transient metabolic acidosis can occur in TS patients, often related to comorbidities, it is not a typical feature. It’s crucial to recognize that the stress-induced changes from intravenous isoprenaline may not be exclusive to RWMA or TS, as isoprenaline induces metabolic changes and histological alterations in all rats, regardless of RWMA development^3^. This suggests that isoprenaline triggers broader systemic effects, which should be considered when interpreting results from similar models^2^. The significance of the glucose difference at 6 hours for RWMA development remains unclear. Nevertheless, associations between fluctuations in blood glucose, insulin, catecholamine release, and myocardial stunning have been reported^28,29^. The TS model demonstrated a comprehensive complication profile matching human clinical presentations, bridging an important gap between experimental and clinical observations. Both mural and protruding thrombi were recorded at a 1.4% incidence, comparable to the 2.2%^30^ and 1.3%^6^ reported in patients. Thrombus formation occurred only in rats with akinetic or dyskinetic segments, emphasizing the critical role of RWMA in TS and mirroring the conditions that promote thrombus formation in humans, such as blood stasis, endothelial injury, and hypercoagulability. The implications of thrombus formation were not studied, as the rats were euthanized upon detection. VT/VF prevalence in TS patients ranges from 2–10%^31^, similar to rates seen in acute myocardial infarction^32^. However, while up to 100% of rats in myocardial infarction models develop VT/VF within the first 60 min^33,34^, the TS model shows a significantly lower incidence of 6.1%. Heart failure is the leading cause of death during the acute phase of both TS and MI, accounting for the majority of in-hospital mortality in clinical settings and deaths in experimental models. The mortality rate in the TS model, including previous data^3^, stands at 7.9%, which is notably lower than the 70% observed in untreated MI models^35^. These observations occur despite the greater extent of akinesia in TS, suggesting different underlying mechanisms. Factors such as enhanced hemodynamic stability, reduced severity of ischemic injury, and greater arrhythmia resistance in TS may contribute to these differences. Collectively, this supports the hypothesis that mechanisms other than severe ischemia play a significant role in the extensive, reversible akinesia observed in our animal model.

Recent reports have linked higher temperatures to increased TS incidence, with a seasonal pattern showing more TS cases during summer^4^. This has been postulated to be related to increased catecholamine levels^36^. Our study revealed a previously unrecognized relationship between body temperature and post-stress RWMA, demonstrating a stepwise increase in akinesia incidence with rising body temperatures. Heart rate was also significantly affected, indicating that temperature modulates the cardiovascular response to catecholamine administration. This modulation could be related to changes in metabolic rate, biochemical reactions, beta-adrenergic responsiveness, or sympathetic nervous system. Despite some rats developing apical ballooning even in hypothermic conditions, and case reports describing TS under similar circumstances^37–39^, our findings suggest that stress-induced RWMA is temperature-dependent. Further research is needed to understand the factors influencing RWMA development post-stress fully.

Several limitations of this study should be acknowledged. The model’s translational validity has inherent constraints. The catecholamine-centred approach with isoprenaline, while effectively inducing the TS phenotype, produces broader systemic effects and may not fully capture the complex, multifactorial nature of human TS. Additionally, the use of healthy rats does not account for pre-existing comorbidities often present in patients. Methodological limitations include the retrospective nature of our REDCap database analysis, which, while maximizing value from previous experiments and adhering to 3R principles, was not powered for specific hypothesis testing. Imaging procedures conducted under anaesthesia likely influenced functional measurements despite using minimal necessary doses. The assessment of RWMA and systolic cardiac function through LVAI and FAC measurements has inherent limitations due to monoplane quantification in the long-axis view. The use of male rats limits generalizability to both sexes. While our model replicates several key features observed in TS patients—suggesting it captures fundamental pathophysiological mechanisms rather than merely superficial similarities—it lacks detailed exploration of molecular and immunological mechanisms.

## Conclusion

Transient RWMA after stress are a hallmark of experimental and clinical takotsubo syndrome. This study offers a detailed exploration of RWMA, emphasizing the crucial roles of contractile function and timing, and charts its development, recovery, and potential as a diagnostic and prognostic tool. Our results demonstrate that body temperature significantly impacts RWMA development and that repeated catecholaminergic surges can exacerbate these abnormalities, possibly delaying recovery and increasing mortality risk. The small-animal model accurately mirrors key aspects of human TS, serving as a valuable tool for advancing research and linking preclinical findings with clinical applications. Since no therapy exists for TS, this study highlights the importance of controlling body temperature and limiting adrenergic drug use in managing TS. Our findings also reveal a delayed adaptive response that mitigates stress-induced RWMA during recovery, offering a potential pathway for developing therapeutic interventions.

## Electronic supplementary material

Below is the link to the electronic supplementary material.

Supplementary Material 1

Supplementary Material 2

Supplementary Material 3

Supplementary Material 4

Supplementary Material 5

## Data Availability

The data underlying this article are available in the article and its online supplementary material.
