# Supplementary material for: Temperature and repeated catecholamine surges modulate regional wall motion abnormalities in a rodent takotsubo syndrome model
Source: Sci Rep. 2025 Jan 31;15:3876. doi: 10.1038/s41598-025-88410-9 (PMC11785725; doi:10.1038/s41598-025-88410-9)
Supplement: Supplementary file 5 — Supplementary Material 5 [file 41598_2025_88410_MOESM5_ESM.docx]

**Supplementary file**

**Supplementary Figure 1.** The natural course of the Takotsubo syndrome model in short axis view with m-mode. Normal contractility can be seen at baseline. At 6 hours TS is verified, the contractility at the base (3 mm from mitral-valve plane) is relatively preserved while at the apex (9mm from mitral-valve plane) the characteristic circumferential pattern, considered a hallmark of TS is noted. The dysfunction in apex is later recovered at 72 hours. **B)** The volumetry methodology involved the fixed incremental short-axis views at 3mm steps from the valve plane down to 9mm depth. At each level, the cross-sectional areas were measured, and the total volume was calculated by combining different geometric shapes: a cylinder from the valve plane to 3mm, a truncated cone from 3mm to 6mm, another truncated cone from 6mm to 9mm, and finally a cone from 9mm to the apex tip. These measurements were performed in both end-systole and end-diastole to enable volume calculations at both cardiac phases.

**Supplementary Figure 2.** Strain-derived akinetic segment. Strain analysis of a takotsubo heart at 6 hours. Note apical akinesia extending to mid segments, here with opposite deformation within the left ventricle. The akinetic segment in strain analysis was measured as the region with <5% radial strain at time of aortic valve closure (highlighted with double arrow (<-->) for clarity). Below figure highlights the time to peak radial strain and peak radial strain for the posterior mid, which in this segment also equals to the post-systolic radial strain. Aortic valve closure represents the time point for end-systolic strain and separates the systolic phase from the post-systolic phase. The highest values for both phases were obtained for peak-systolic strain and post-systolic strain, respectively. The proportion of strain occurring post-systole, termed the post-systolic radial strain ratio, was calculated as the ratio of post-systolic radial strain to peak radial strain. **B) (LEFT)** Change in longitudinal strain measured in LAX view. Rats with the TS phenotype had a significantly reduced longitudinal strain (mean + SE). Paired t-test. ***; P<0.001. **(RIGHT)** Length of the transition zone in mm. The transition zone was defined as the area between <5% and >15% radial strain at the aortic valve closure. **C)** Definition of aortic valve closure. Strain analysis of a rat heart at baseline. Aortic valve closure was defined in the dV/dt-graph when start of isovolumetric relaxation begin. Here highlighted by the first red line, second red line shows its end. Above heat maps displays radial strain over two cardiac cycles. **D)** The transition zone from akinetic to non-akinetic tissue as seen in the strain analysis. Radial strain at aortic valve closure is shown.

**Supplementary Figure 3.** **A)** Change in global WMSI over time, calculated as an average of 17 segments in the left ventricle, mean + SE. Results presented as one-way repeated measure with Greenhouse-Geisser correction. P-values adjusted using Bonferroni procedure, n = 12. ns; not significant. ***P < 0.001 ***P < 0.001 vs. 90 min. **B)** Kaplan-Meier survival curves display recovery times across different heart regions, with shaded areas indicating confidence intervals. Differences between regions were statistically evaluated using log-rank tests. A Cox proportional hazards model, with the apex as the reference category, was used to calculate hazard ratios (table). The highest likelihood of recovery was observed in the base region, followed by the mid region. Regions recovered between 3-30 days were capped at 4 days. **C)** Temporal changes in WMSI across different heart regions (base, mid, apex), mean + SE. Regional WMSI was determined by averaging WMSI for each region. The mid region displays temporal fluctuations, characterized by an increase in WMSI at 6 hours followed by a decrease at 24 hours. In contrast, the apex region shows consistently elevated WMSI levels during this period. Data were analysed using two-way repeated measures ANOVA, with Bonferroni-adjusted P-values, n = 12 per group. **P < 0.01, ***P < 0.001 apex vs. mid; ¤P < 0.05, ¤¤¤P<0.001 vs. 90min (apex); #P < 0.05, ##P < 0.01, ###P < 0.001 vs. 90min (mid). **D)** Change in WMSI over segments across different time points, presented as mean + SE.

**Supplementary Figure 4.** Segmental end-systolic radial strain, post-systolic radial strain, and post-systolic radial strain ratio in TS hearts (at the 6-hour time point) and at baseline. Profound impairment in myocardial contraction is observed at apex and mid. However, the elevated post-systolic radial strain and higher post-systolic radial strain ratios in the mid and apex regions during the acute phase of TS suggest the presence of viable myocardium with ongoing but delayed contraction. Results presented as Friedman test and the Wilcoxon signed-rank test. Benjamini-Hochberg p-value adjustments. ns; not significant. *; P<0.05. **; P<0.01. ***; P<0.001.

**Supplementary Figure 5.** Bland-Altman and Scatter Plots with Correlations. **A & B)** FAC and longitudinal strain for measuring systolic function generally agree and display a strong correlation (r = 0.796, p < 0.001, n = 81), although with some variability and a tendency for increased differences at higher average values. **C & D)** FS and radial strain for measuring apical contractility have a very strong correlation (ρ = 0.846, p < 0.001) and show favourable agreement at low strain levels, whereas greater variability is observed at higher values (n = 48). **E & F)** Visual assessment of akinetic segments correlates well with segments identified using strain analysis. A cut-off value of 5% at the time of aortic valve closure (ρ = 0.778, p < 0.001) or 10% (ρ = 0.768, p < 0.001) provides similar results. The visual assessment tends to be higher on average, with indications of proportional bias (n = 24). Inner dashed lines depict the mean (bias), and outer dashed lines represent the 95% limits of agreement. Note that longitudinal strain values have been adjusted to align with the direction of FAC measurement for enhanced interpretability. Spearman ´s or Pearson´s correlation coefficient.

**Supplementary Figure 6**. **A)** ROC curves portray the ability of LVAI, left, and FAC, right, in predicting mortality (AUC: 0.8227 and 0.9663 respectively). Measurements performed at first echo time-point after induction. Results are presented using logistic regression analysis as ROC curve and area under the curve. For akinesia Odds Ratio (OR) 1.13; 95% confidence interval (CI) 1.07-1.20. For FAC (%) OR 0.79; 95% CI 0.69-0.87. **B)** Correlogram of different measure of cardiac function. Strong correlation between FAC and LS, EF, and LVAI. Rats with no akinesia are excluded. *n* = 74 observations. Pearson correlation. **C)** Rats that did not develop any akinesia or regional wall motion abnormalities after catecholamine challenge (mean + SE). The hearts seem to either respond by increasing cardiac function (red circle), stay unchanged (green circle), or decrease (blue circle) 2 hours after receiving catecholamine. n = 12. **D)** Changes in blood glucose over time. Rats without apical akinesia had higher levels compared to TS rats at 6 hours (mean + SE). Analysed using repeated measure mixed model ANOVA, with time as repeated factor and TS (yes/no) as treatment factor. *n* = 6/group. *P < 0.05 no-TS vs. TS.

**Supplementary Figure 7.** Categorised case plots showcasing the differences in akinesia and cardiac function between the first and second stressor. Individual observations are color-coded. **A)** 6 hour restress. Three rats did not develop any akinesia after the first stress, but two of them developed after the second. *n* = 14. **B)** 24 hour restress. One rat did not develop any akinesia after the first stress, this did not change after the restress. *n* = 25. **C)** 30 day restress, only one rat had more akinesia after the second round compared to the first. Two rats did not develop any akinesia after the first challenge, and one of them developed akinesia in the second round. *n* = 22.

**Supplementary Figure 8.** Complementary echocardiographic variables from the 6h restress study. Results presented as one-way repeated measure with Greenhouse-Geisser correction. P-values adjusted using Bonferroni procedure. ns; not significant. *; P<0.05. **; P<0.01. ***; P<0.001. SV; Stroke volume. EDV; End-diastolic volume. ESV; End-systolic volume. CO; Cardiac output.

**Supplementary Figure 9. A)** Kaplan Meier analysis showing the probability of being free from any arrhythmia (AV-block, VT, or VF). **B)** Cumulative incidence of all arrhythmia occurrence stratified by type; VT, VF, or AV-block. The first 20 minutes of isoprenaline infusion was a particular sensitive phase for arrhythmia occurrence. **C)** A typical non-sustained Torsade de Pointes that occurred in some rats and was defined as a VT (i). Premature ventricular beats with full compensatory pauses were prevalent in all rats in varying extent (ii/iii). **D)** Scatterplot of FAC over LVAI at 4-7 hours post TS-induction. Only observations with LVAI >0% and measured FAC were included from the REDCap database. 16% (28 out of 173) had an FAC above 45% (red line), while 9% (16 out of 173) had an FAC above 50%.

**TABLE LEGENDS**

**Supplementary Table 1**. Overview of significant arrhythmia types in the TS model. n = 131. Average recording time = 70 min.

**Supplementary Table 2**. Blood gas analysis in rats with takotsubo syndrome during the first 24 hours. Normal values are seen at 6 hours. During its development however, a transient metabolic acidosis with increase in lactate that is respiratory and metabolic compensated is noted. One-way repeated measures ANOVA, with time point as the repeated factor. P-values are adjusted using Bonferroni multiple comparison procedure. Comparisons were performed if Greenhouse-Geisser <0.05. n = 9/timepoint. **Bolded**; p < 0.05 vs baseline.

**VIDEO LEGENDS**

**Supplementary Video 1.** How LVAI and FAC is measured. LVAI measurements were done in diastole (“Traced distance”) while FAC both in diastole (LVEDA, denoted “Area” in the image) and systole (LVESA). The diastolic image with measurements is shown above. The blue tracings are done in the Vevo Lab software, the white dashed-lined has been added to highlight the akinetic segment for clarity. Corresponding cine-loop can be seen below. The R-R cycle with best visible endocardial border and maximum distance between the aortic valve and the cardiac apex was chosen for analysis.

**Supplementary Video 2**. How the total fractional shortening is measured at each segment. Its corresponding basal cine loop is shown above. The apical cine-loop is shown for comparison.

**Supplementary Video 3**. Strain analysis of 6-hour TS heart. Longitudinal strain was applied to the entire length of the endomyocardial line

**Supplementary Video 4.** Development of a protruding apical thrombus in a rat with apical akinesia post isoprenaline challenge.

**Supplementary Table 1**

Blood gas analysis in rats with Takotsubo syndrome during the first 24 hours

|  | **Baseline (N=9)** | **30 min (N=9)** | **90 min (N=9)** | **6 hours (N=9)** | **24 hours (N=9)** |
| --- | --- | --- | --- | --- | --- |
| **pH** |  |  |  |  |  |
| Mean (SD) | 7.36 (0.0259) | 7.38 (0.0337) | 7.37 (0.0319) | 7.37 (0.0204) | 7.41 (0.0459) |
| Median [Min, Max] | 7.37 [7.30, 7.38] | 7.38 [7.30, 7.42] | 7.37 [7.34, 7.43] | 7.38 [7.33, 7.40] | 7.42 [7.31, 7.46] |
| **pCO2 (kPa)** |  |  |  |  |  |
| Mean (SD) | 7.08 (0.478) | **5.52 (0.957)*** | 5.97 (0.802) | **6.01 (0.511)*** | **5.72 (0.532)*** |
| Median [Min, Max] | 7.21 [6.31, 7.94] | 5.45 [4.67, 7.94] | 6.13 [4.47, 6.94] | 6.16 [5.14, 6.54] | 5.58 [5.32, 7.01] |
| **pO2 (kPa)** |  |  |  |  |  |
| Mean (SD) | 13.6 (4.15) | 11.7 (2.59) | 33.4 (19.4) | **41.6 (12.8)*** | 39.4 (17.2) |
| Median [Min, Max] | 11.6 [9.25, 19.7] | 10.5 [9.22, 16.9] | 39.3 [9.54, 56.2] | 42.4 [18.8, 63.5] | 50.3 [13.1, 56.7] |
| **Na+** |  |  |  |  |  |
| Mean (SD) | 143 (2.30) | 143 (1.58) | 141 (2.06) | **140 (1.74)*** | 140 (2.00) |
| Median [Min, Max] | 144 [139, 147] | 143 [141, 146] | 142 [139, 145] | 139 [136, 142] | 141 [137, 143] |
| **K+ (mmol/L)** |  |  |  |  |  |
| Mean (SD) | 4.04 (0.309) | 3.88 (0.277) | 3.80 (0.480) | 4.06 (0.490) | 4.38 (0.533) |
| Median [Min, Max] | 3.90 [3.70, 4.70] | 3.90 [3.50, 4.40] | 3.70 [3.10, 4.60] | 4.20 [3.40, 4.80] | 4.50 [3.40, 5.00] |
| **Ca++ (mmol/L)** |  |  |  |  |  |
| Mean (SD) | 1.44 (0.0547) | 1.39 (0.0367) | 1.32 (0.0758) | **1.33 (0.0459)*** | 1.36 (0.0314) |
| Median [Min, Max] | 1.41 [1.38, 1.52] | 1.38 [1.35, 1.46] | 1.36 [1.22, 1.41] | 1.33 [1.21, 1.37] | 1.38 [1.30, 1.40] |
| **Cl- (mmol/L)** |  |  |  |  |  |
| Mean (SD) | 104 (1.93) | 108 (2.83) | 106 (2.60) | 106 (4.30) | 106 (2.78) |
| Median [Min, Max] | 103 [101, 106] | 109 [104, 113] | 105 [104, 111] | 103 [102, 113] | 106 [102, 111] |
| **Glu (mmol/L)** |  |  |  |  |  |
| Mean (SD) | 9.09 (0.578) | 8.99 (0.884) | 9.25 (1.39) | 9.43 (1.74) | 10.0 (1.17) |
| Median [Min, Max] | 9.10 [8.40, 10.3] | 8.90 [7.80, 10.4] | 9.40 [7.30, 11.1] | 8.80 [7.60, 12.4] | 10.1 [8.40, 11.3] |
| **Lac (mmol/L)** |  |  |  |  |  |
| Mean (SD) | 1.25 (0.300) | **4.71 (1.50)*** | 2.59 (1.02) | 1.43 (0.935) | 2.38 (0.652) |
| Median [Min, Max] | 1.19 [0.88, 1.74] | 4.82 [1.67, 6.75] | 2.69 [0.82, 4.19] | 1.12 [0.30, 3.33] | 2.40 [1.34, 3.07] |
| **Crea (μmol/L)** |  |  |  |  |  |
| Mean (SD) | 27.0 (0) | 33.2 (7.41) | 49.4 (21.5) | 45.1 (22.7) | 37.9 (13.4) |
| Median [Min, Max] | 27.0 [27.0, 27.0] | 32.0 [27.0, 46.0] | 49.0 [28.0, 91.0] | 39.0 [27.0, 101] | 35.0 [27.0, 66.0] |
| **Hct (L/L)** |  |  |  |  |  |
| Mean (SD) | 0.37 (0.011) | 0.37 (0.014) | 0.35 (0.013) | 0.36 (0.025) | 0.36 (0.020) |
| Median [Min, Max] | 0.38 [0.35, 0.39] | 0.37 [0.35, 0.39] | 0.35 [0.34, 0.38] | 0.36 [0.33, 0.40] | 0.36 [0.33, 0.40] |
| **cHCO3- (mmol/L)** |  |  |  |  |  |
| Mean (SD) | 29.7 (1.30) | **24.2 (2.51)*** | **26.0 (2.63)*** | 26.3 (3.12) | **27.3 (1.75)*** |
| Median [Min, Max] | 29.5 [27.8, 31.6] | 24.0 [19.8, 29.4] | 26.6 [22.1, 29.2] | 27.7 [21.1, 29.4] | 27.0 [24.2, 29.6] |
| **cSO2 (%)** |  |  |  |  |  |
| Mean (SD) | 96.4 (2.35) | 95.9 (1.94) | 98.2 (2.62) | **99.8 (0.310**)* | 99.5 (1.06) |
| Median [Min, Max] | 95.8 [93.0, 99.2] | 95.5 [93.3, 98.4] | 99.9 [93.4, 100] | 99.9 [99.0, 100] | 100 [96.8, 100] |
| **AGap (mmol/L)** |  |  |  |  |  |
| Mean (SD) | 10.9 (1.96) | 11.1 (2.76) | 9.56 (2.83) | 8.44 (1.94) | 8.11 (1.76) |
| Median [Min, Max] | 11.5 [8.00, 13.0] | 10.0 [8.00, 16.0] | 9.00 [6.00, 15.0] | 9.00 [6.00, 11.0] | 8.00 [6.00, 11.0] |
| Glu: Glucose. Lac: Lactate. Crea: Creatinine. Hct: Hematocrit. AGap: Anion Gap. **Bolded*:** p < 0.05 vs baseline, repeated measure ANOVA with Bonferroni correction. | | | | | |

| **Arrhythmia Type** | **Number of Rats Affected (% of total)** | **Incidence Rate (per hour)** | **Mean Episodes in Affected Rats** | **Mean Duration Per Episode (seconds)** | **Range of Durations (seconds)** | **Deaths Associated** |
| --- | --- | --- | --- | --- | --- | --- |
| Ventricular Tachycardia (VT) | 8 (6.1%) | 0.100 | 2.0 | 5.3 | 0.5 - 22 | 0 |
| Ventricular Fibrillation (VF) | 5 (3.8%) | 0.031 | 1.0 | 27.0 | 4 - 50 | 3 |
| AV-Block | 4 (3.1%) | 0.025 | 1.0 | N/A | N/A | 4 |

**Supplementary Table 2**

N/A; not applicable.

**Supplementary Figure 1**


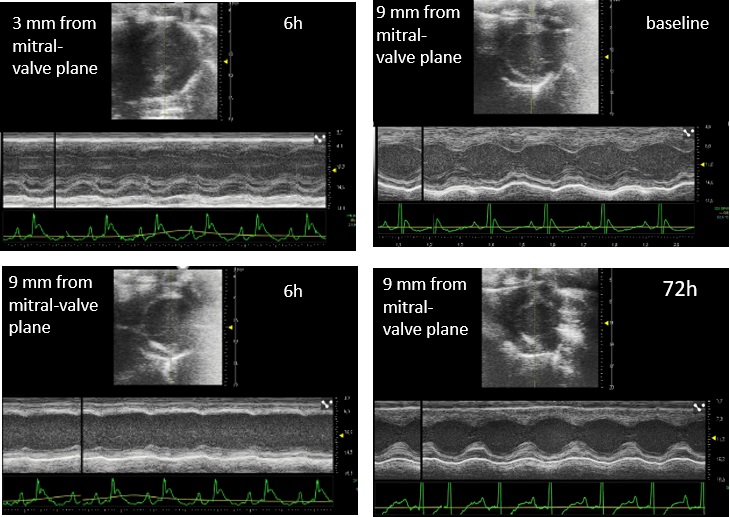


**B)**

**
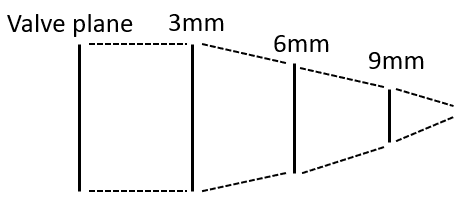
**

**Supplementary Figure 2**

**
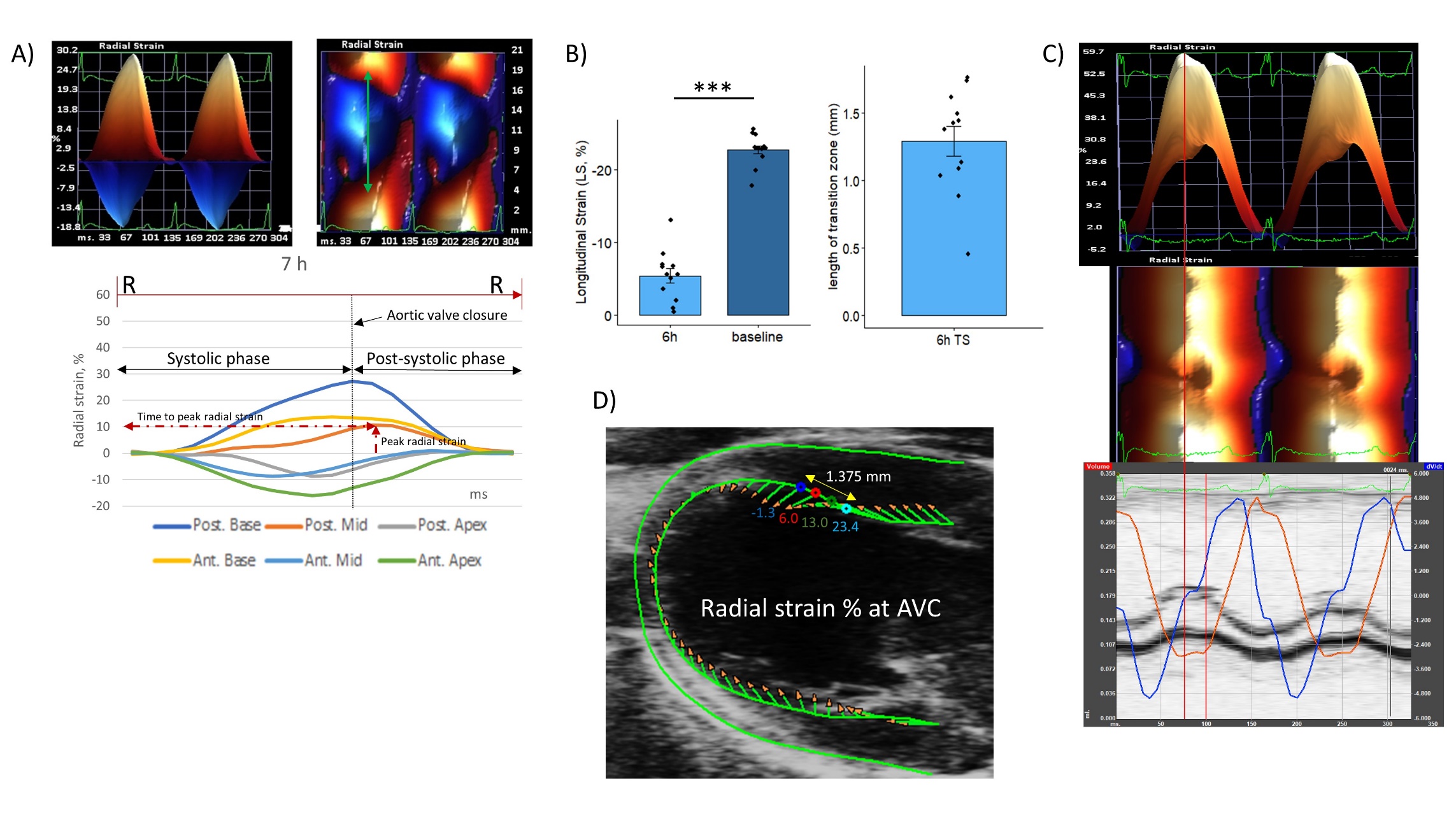
**

**Supplementary Figure 3**

**
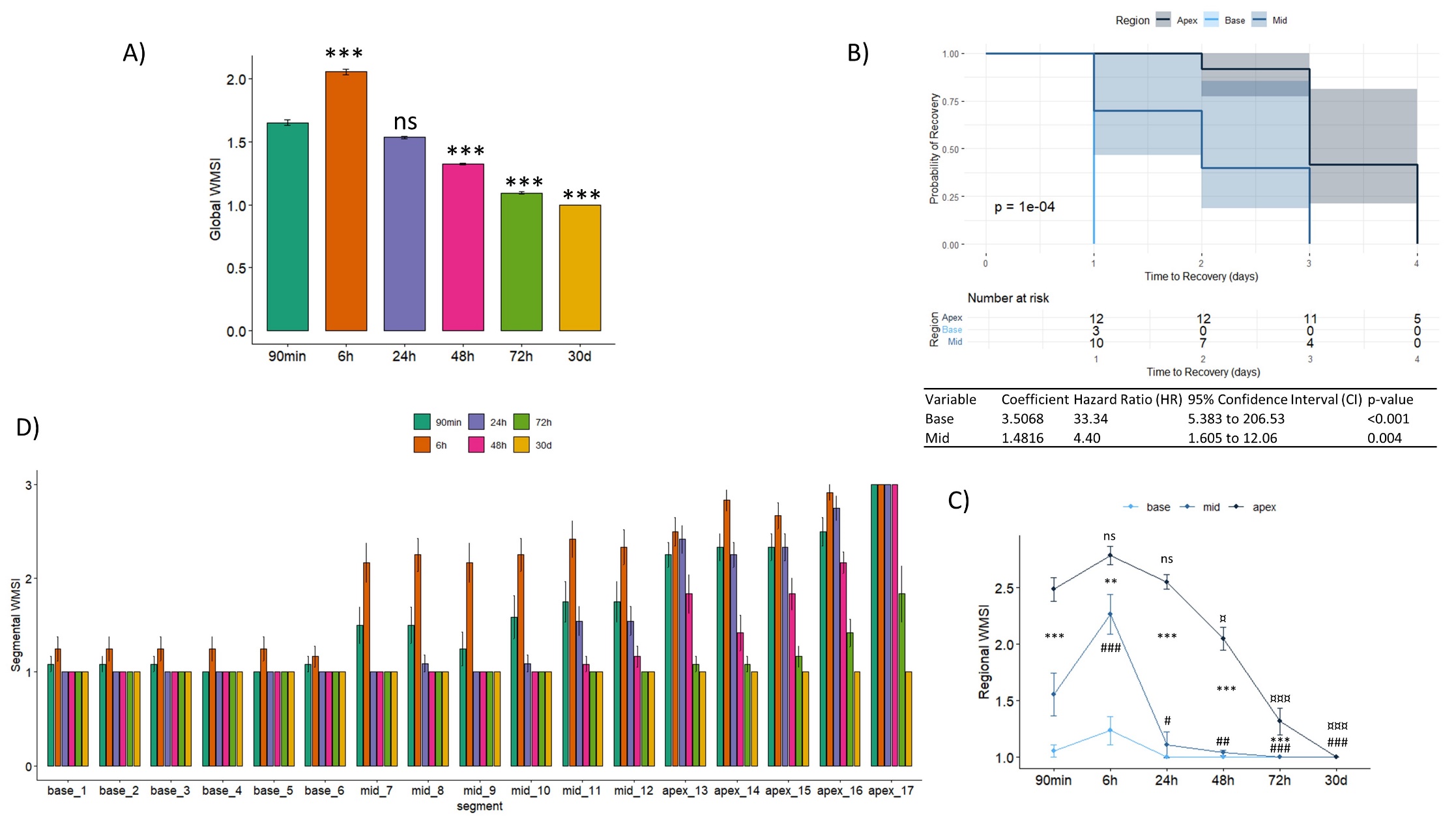
**

**Supplementary Figure 4** **
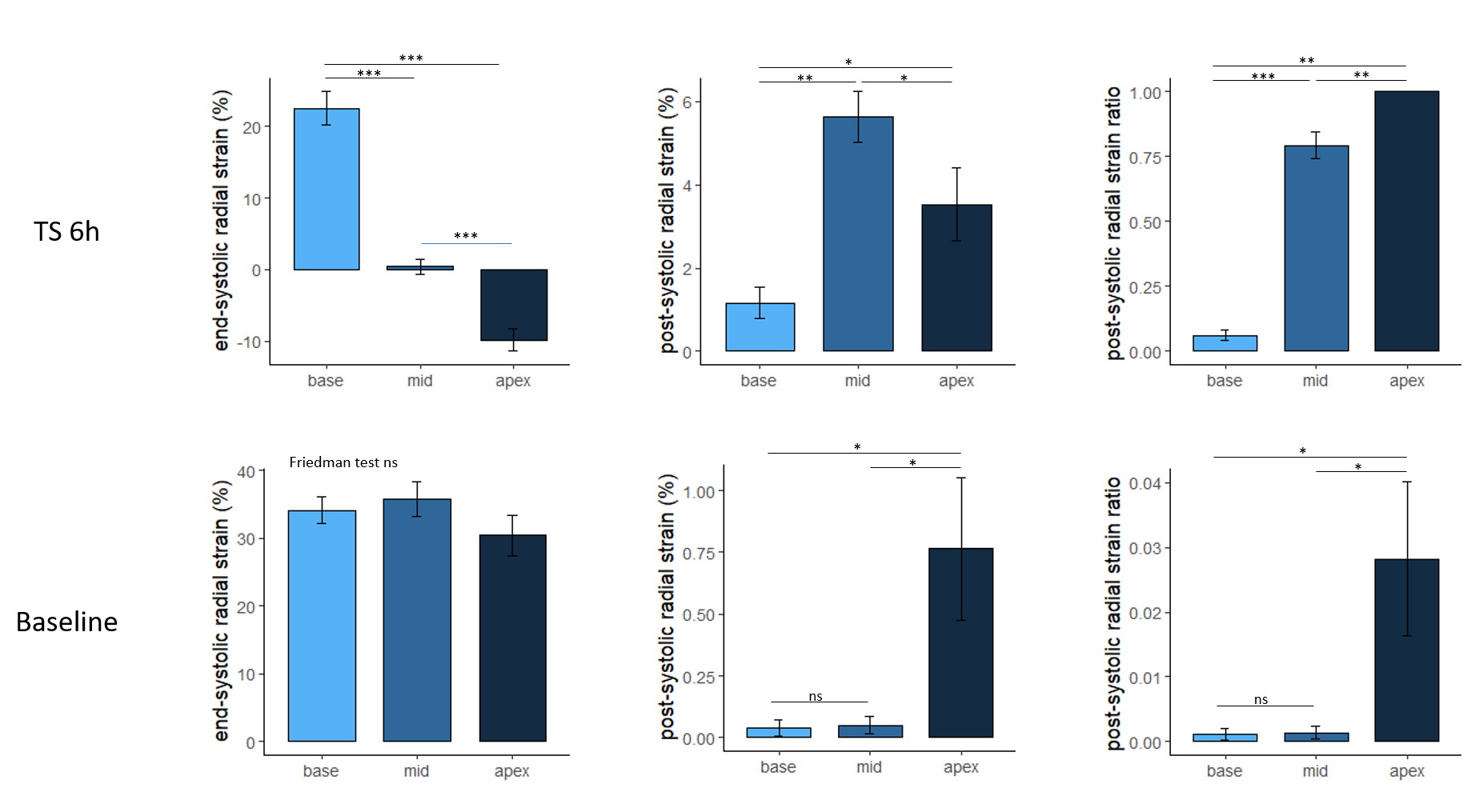
**

**Supplementary Figure 5**

*
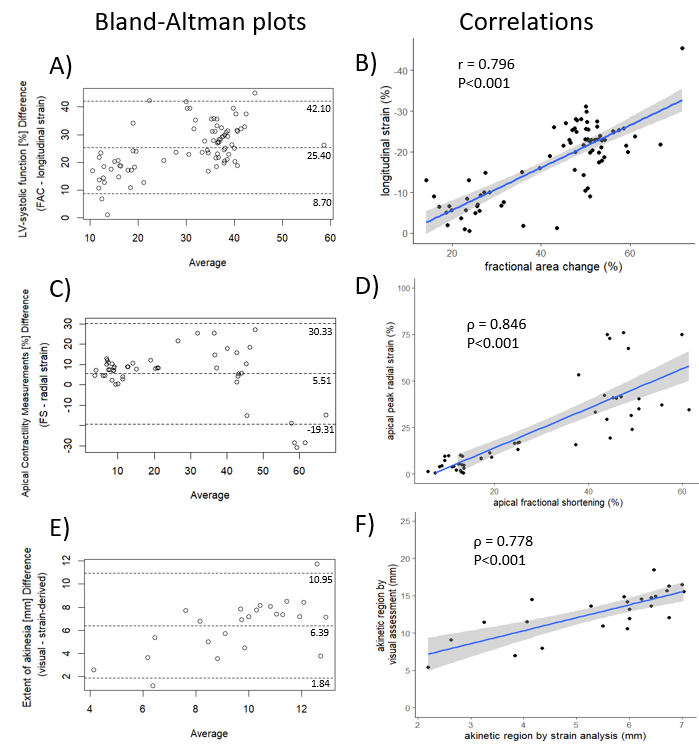
*

**Supplementary Figure 6**

**
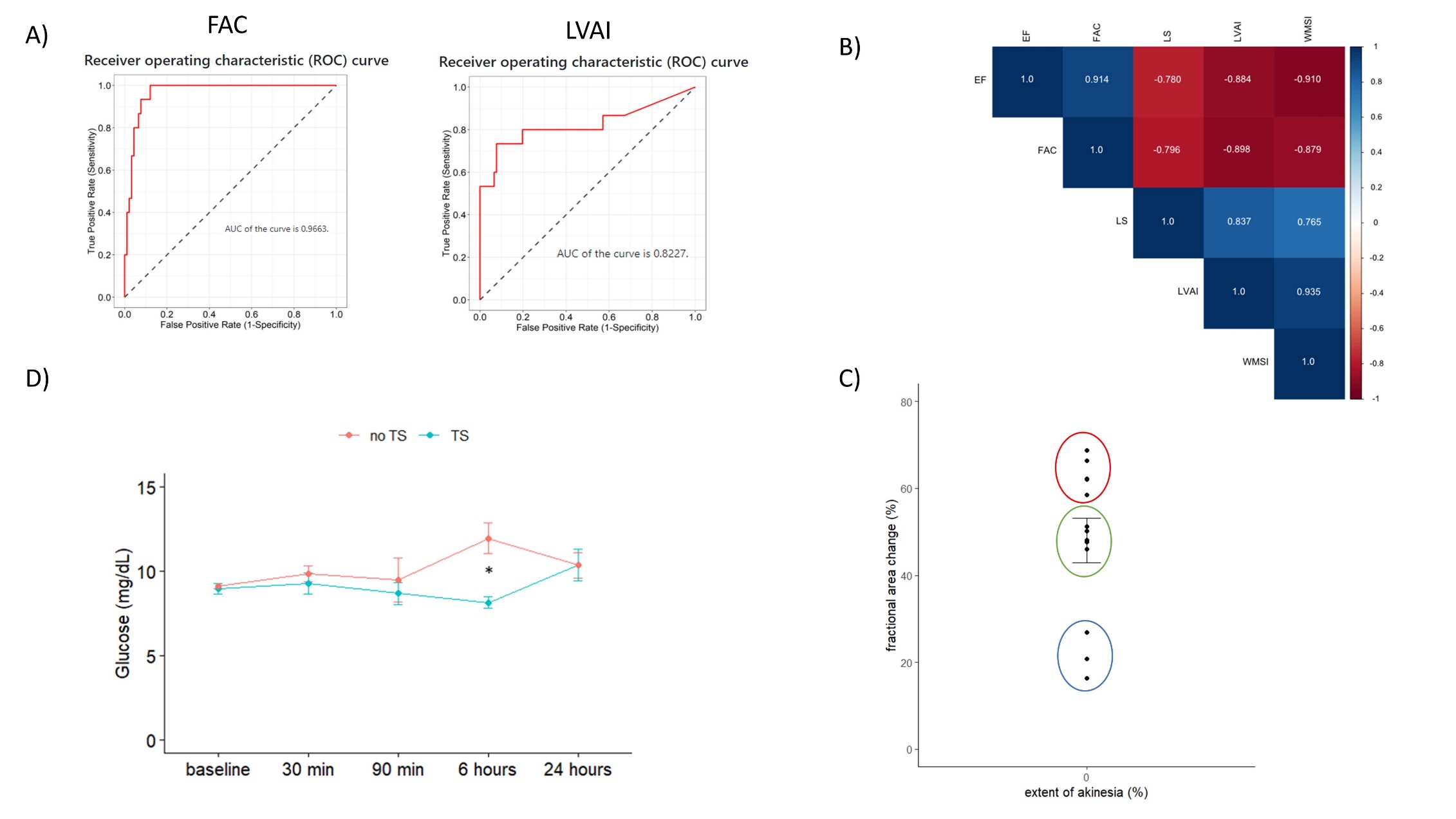
**

**Supplementary Figure 7**

**
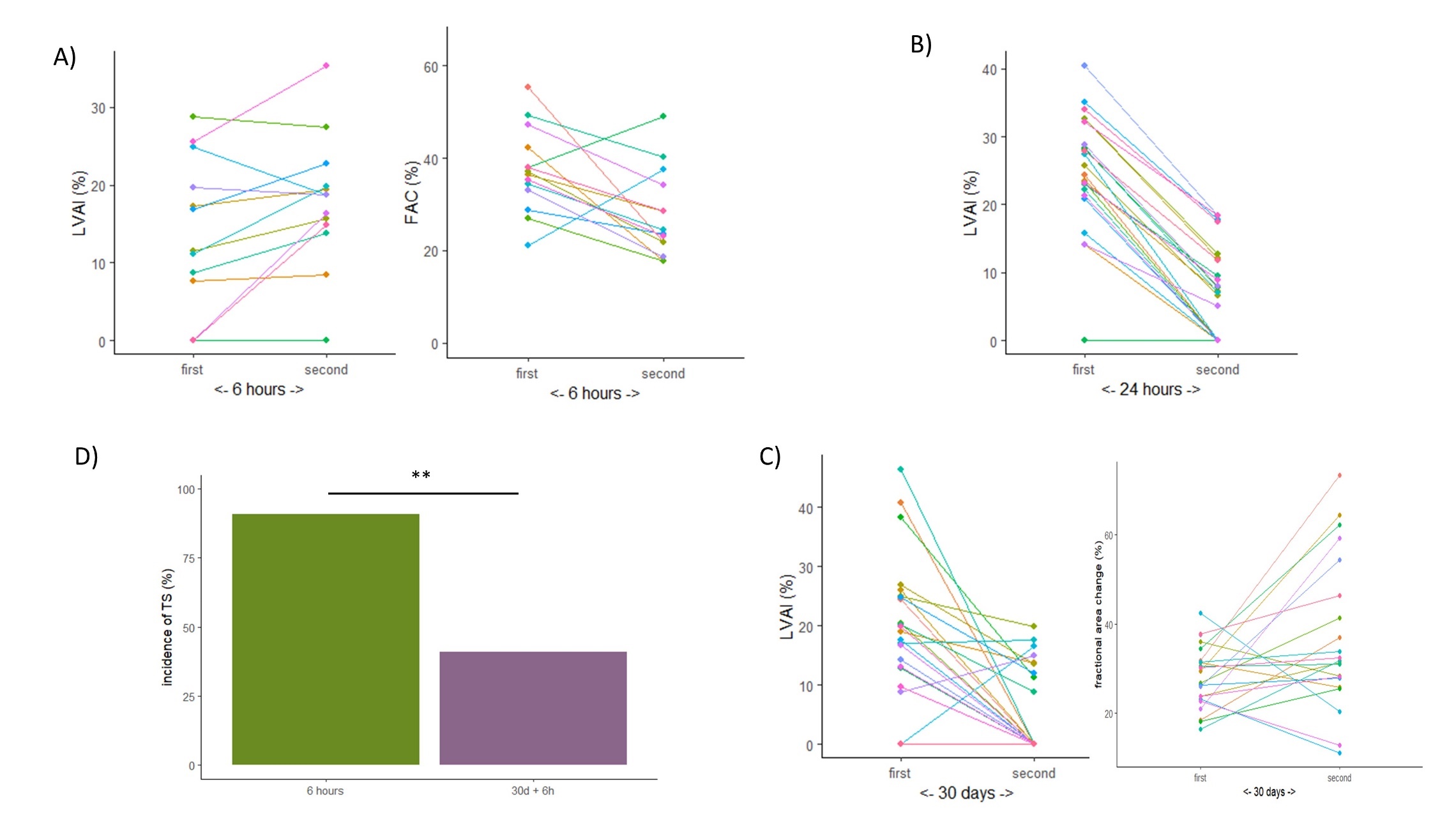
**

**Supplementary Figure 8**

*
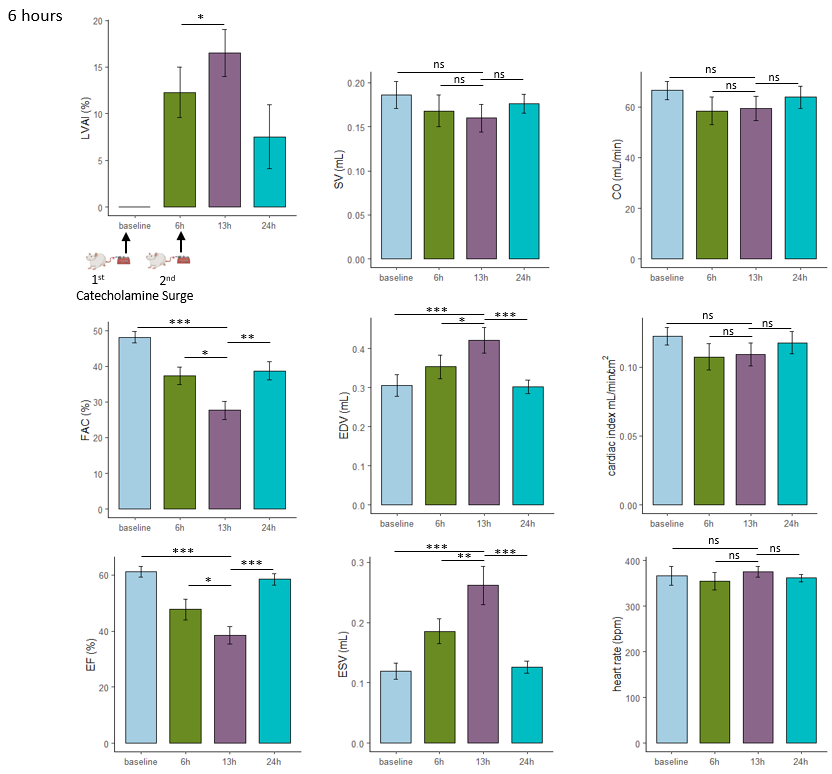
*

**Supplementary Figure 9
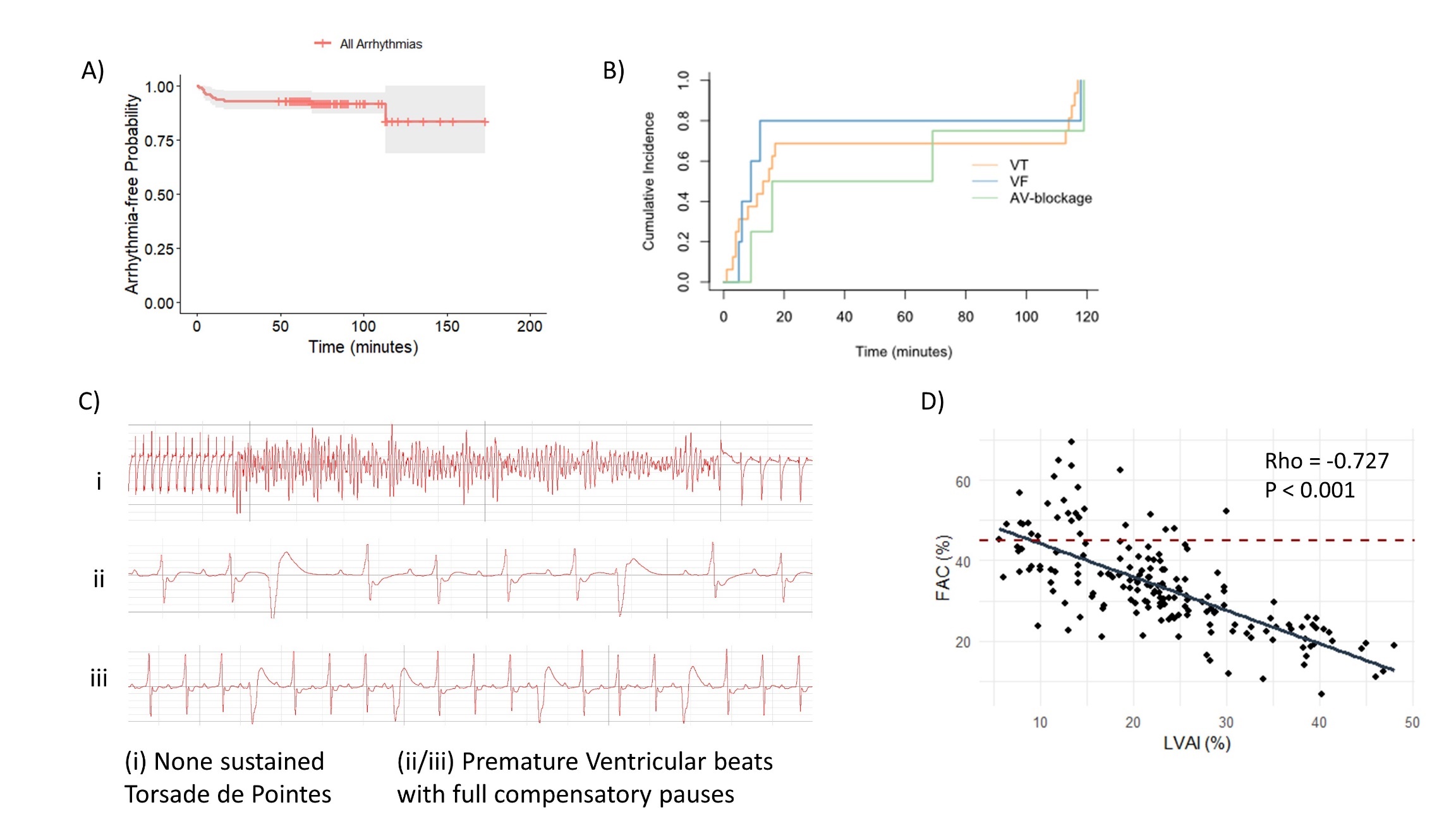
**

*Statistical analysis*

Randomization was done by generating random numbers. The randomization sheet was blinded to the researchers and uploaded to the REDCap electronic data capture tools^15^, where all study data was collected. The data visualizations and statistical analysis were performed using R 4.2.0, IBM SPSS Statistics 27, and InVivo Stat v4.2.0. The sample size was calculated using G*Power 3.1.9.7 with data from previous studies (power = 0.8, effect size η^2^ = 0.19, alpha level = 0.05). The animal was the experimental unit for all data analyses, and the day was used as a blocking factor. Parametric assumptions were checked through visual plots, normal probability plots of residuals, and predicted vs. Externally Studentised Residuals plots. Comparison of akinesia between groups were done through paired two sample t-test for 2 conditions (6h restress, 30d restress) and the non-parametric gateway test Kruskal-Wallis for ≥2 conditions (temperature study), with a post-hoc pairwise comparison using Wilcoxon rank sum exact test and Bonferroni procedure for p-value adjustment. For repeated measures where more than two factors were present the two-way repeated measure ANOVA was used (blood gas analysis). One-way repeated measure when only 1 factor was present (restress studies, blood gas analysis), and Greenhouse-Geisser <0.05 was considered significant. Pearson test (r) or Spearman’s rank test (ρ) was used for correlations. Bland-Altman plots was performed to assess the agreement between two methods. To estimate the predicting ability of LVAI and FAC for mortality, a logistic regression analysis was performed and presented with corresponding receiver operator characteristics (ROC) curve and area under the curve (AUC) value. To estimate the predictive ability of LVAI on recovery time, we employed a Generalized Additive Model (GAM) with weighted least squares. This approach was chosen to account for potential non-linear relationships and heteroscedasticity observed in the residuals of simpler linear models. The GAM was specified with the log-transformed recovery time as the response variable and LVAI as the predictor variable. The model was fitted using the mgcv (1.9.1) package in R, which allows for smooth terms in the predictor variables to capture potential non-linear effects. The formula used was: log(Recovery) ~ s(LVAI), where s() denotes a smooth term. Residuals from the initial GAM fit were used to calculate weights as the inverse of the absolute residuals. The weighted model was then refitted using these calculated weights to address heteroscedasticity. Model performance was presented as adjusted R-squared and root mean square error. Kaplan–Meier analysis with log‐rank testing was applied for survival analysis. Cluster analysis was conducted using agglomerative hierarchical clustering with the Ward linkage method, and data was normalized using Z-score normalization. The number of clusters was determined from the dendrogram, and the Euclidean distance metric was employed in the clustering process. Fischer exact t-test was used to compare incidence. Extended Mantel-Haenszel chi square was used to calculated trend of TS incidence in the temperature study.
